# Human Embryonic Stem Cell–derived Neural Crest Cells Promote Sprouting and Motor Recovery Following Spinal Cord Injury in Adult Rats

**DOI:** 10.1177/0963689720988245

**Published:** 2021-01-31

**Authors:** Iwan Jones, Liudmila N. Novikova, Mikael Wiberg, Leif Carlsson, Lev N. Novikov

**Affiliations:** 1 59588 Umeå Center for Molecular Medicine, Umeå University, Umeå, Sweden; 2 Department of Integrative Medical Biology, Umeå University, Umeå, Sweden; 3 Department of Surgical and Perioperative Science, Section of Hand and Plastic Surgery, Umeå University, Umeå, Sweden

**Keywords:** hESCs, neural crest cells, spinal cord injury, transplantation, vertical cylinder test, motor recovery

## Abstract

Spinal cord injury results in irreversible tissue damage and permanent sensorimotor impairment. The development of novel therapeutic strategies that improve the life quality of affected individuals is therefore of paramount importance. Cell transplantation is a promising approach for spinal cord injury treatment and the present study assesses the efficacy of human embryonic stem cell–derived neural crest cells as preclinical cell-based therapy candidates. The differentiated neural crest cells exhibited characteristic molecular signatures and produced a range of biologically active trophic factors that stimulated *in vitro* neurite outgrowth of rat primary dorsal root ganglia neurons. Transplantation of the neural crest cells into both acute and chronic rat cervical spinal cord injury models promoted remodeling of descending raphespinal projections and contributed to the partial recovery of forelimb motor function. The results achieved in this proof-of-concept study demonstrates that human embryonic stem cell–derived neural crest cells warrant further investigation as cell-based therapy candidates for the treatment of spinal cord injury.

## Introduction

The majority of spinal cord injuries (SCI) occur at the cervical level, and affected individuals are left with severe physical and mental health challenges due to sensorimotor and autonomic impairment in addition to neuropathic pain^1–3^. Primary physical impact leading to neuroglial and vascular damage, blood-spinal cord barrier disruption, hemorrhage, and ischemia during the initial acute phase of SCI (ASCI) gradually develops into a secondary injury cascade, which can last for several weeks and is characterized by the expansion of tissue damage, neuroglial degeneration, demyelination, and macrophage-mediated axonal dieback^4–6^.

Several months after patients are discharged from hospital they are considered to be in the chronic phase of SCI (CSCI). At this stage the lesion site often contains cysts and cavities and is surrounded by a thick scar with fibrotic and glial components^5,7^. Other characteristic milestones of CSCI are persistent neuroinflammation with suppressed systemic immunity in addition to delayed secondary degeneration of neurons in descending and ascending long spinal tracts^4,8^. The development of effective therapeutic strategies that attenuate the immune response and foster functionally significant de novo axonal regeneration and sprouting following SCI is therefore important to improve the life quality of affected individuals^2,5,9,10^.

Cell transplantation therapies have become a promising preclinical strategy for the treatment of SCI, and various human derived cell types including neural stem cells, Schwann cells, olfactory-ensheathing cells, and mesenchymal stromal cells have been tested both experimentally and clinically as suitable candidates^2,6,10–13^. Several possible mechanisms that underlie the therapeutic effects of cell transplants have been proposed and encompass neuroprotection, the stimulation of axonal regeneration and sprouting in addition to the modulation of the neuroinflammation and remyelination responses^2^. Human embryonic stem cells (hESCs) possess the greatest conceptual potential for the development of novel cell-based SCI therapies since they are infinitely renewable and amenable to molecular manipulation^14^. To this end, hESC-derived neural progenitors have been shown to support functional recovery in animal SCI models in addition to showing promise in human Phase I/II clinical trials^2,10^. However, studies have also demonstrated that neural stem cells can induce deleterious side effects such as allodynia-like hypersensitivity and the formation of ectopic colonies following focal transplantation^15,16^. The identification of hESC-derived transplantation candidates that circumvent these side effects is therefore of significant importance for the development of the future treatment strategies for SCI.

hESC-derived neural crest cells are uniquely qualified as cell-based SCI therapy candidates due to their multipotent capability and ontological similarity to spinal cord stem cells^17–19^. Moreover, our previous study demonstrated that neural crest cells could be rapidly differentiated from hESCs and their transplantation into a peripheral nerve injury model promoted robust sciatic nerve regeneration without tumor formation^20^. The work presented in this study therefore extends on our previous investigation and assesses the efficacy of hESC-derived neural crest cells for the treatment of acute and chronic cervical SCI. The differentiated neural crest cells exhibited characteristic molecular signatures and produced a range of biologically active trophic factors that stimulated the *in vitro* outgrowth of rat primary dorsal root ganglia (DRG) neuronal cultures. Transplantation of the neural crest cells into a rat cervical SCI model promoted remodeling of descending motor tracts and partial motor recovery of forelimb function in both ASCI and CSCI scenarios. The results achieved in our proof-of-concept study therefore demonstrates that hESC-derived neural crest cells warrant further investigation as cell-based therapy candidates for the treatment of SCI.

## Materials and Methods

### hESC Culture

hESCs (H9, WA09, Passages 31 to 41) were cultured on irradiated embryonic fibroblasts isolated from CF-1 mice^21^ in Dulbecco’s modified Eagle’s medium/F12 supplemented with 20% (v/v) KnockOut Serum Replacement, 1% (v/v) nonessential amino acids, 100 mM l-glutamine, 0.1 mM β-mercaptoethanol, 1% (v/v) penicillin–streptomycin (PeSt), and 4 ng/ml basic fibroblast growth factor (bFGF). hESC colonies were enzymatically passaged by collagenase IV treatment as described previously^22^. All hESC culture reagents were purchased from Thermo Fisher Scientific, Stockholm, Sweden.

### Differentiation of Neural Crest Cells from hESCs

Neural progenitor cells were differentiated from hESCs by small molecule inhibition of SMAD and WNT-signaling pathways^23^ and the NGFR^+^ neural crest population was subsequently isolated by magnetic activated cell sorting (MACS) using a CD271 human microbead kit (Miltenyi Biotec Norden AB, Lund, Sweden). The NGFR^+^ neural crest cell population was then seeded on Matrigel-coated culture vessels in N2 media^23^ supplemented with 10 ng/ml bFGF and 20 ng/ml epidermal growth factor (EGF). Neural crest cell cultures were enzymatically passaged onto Matrigel-coated culture vessels by accutase treatment for a maximum of five passages prior to transplantation. A total of three independent differentiation experiments were performed during this study.

### Experimental Animals, SCI Model, and Cell Transplantation

Transplantation experiments were performed on adult (10- to 12-week-old) female Sprague–Dawley rats under isoflurane anesthesia in combination with buprenorphine (Temgesic, Indivior Europe Ltd, Dublin, Ireland; 0.025 mg/kg, subcutaneous). Following dorsal cervical C3–C4 laminectomy on the left side, a small 3 × 2 mm window was created in the bone using Friedman-Pearson Rongeurs with a 0.5 mm cup size. The dura mater was cut longitudinally and the border between the C3 and C4 dorsal root entry zone and the dorsal funiculus was identified prior to being vertically penetrated with a 23G needle. After introducing one blade of a microserrated Vannas spring scissors into the stab wound and the other blade between the lateral surface of the spinal cord and the dura mater, the lateral funiculus and adjacent gray matter were transected from the lateral side. Care was taken to avoid additional damage to the dura mater. This injury interrupts descending bulbospinal motor tracts from the brainstem in addition to ascending sensory and propriospinal pathways. The corticospinal tract in the dorsal column was spared. Immediately after the injury, the animals were randomly divided into three experimental groups: (i) neural crest cell transplantation into acute SCI (ASCI; *n* = 17), (ii) neural crest cell transplantation into chronic SCI (CSCI; *n* = 12), and (iii) time-matched SCI controls with medium injections (*n* = 17). Normal uninjured rats (*n* = 15) served as baseline controls (for details, see Supplemental Table S1). The animals selected for the transplantation into ASCI and matching SCI control animals were mounted into a stereotaxic instrument (see below) and cell injection (ASCI) or injection of a cell medium (SCI control) was begun within 30 min after initial surgery. Transplantation into CSCI was performed 7 weeks postoperatively. In experimental rat models of SCI, the chronic injury period is defined as one that begins at least 4 weeks post-trauma^4^. The animals were anesthetized as described above, the C3–C4 cervical vertebrae were re-exposed and the widow in the vertebrae created during previous laminectomy was carefully extended both rostral and caudal. The position of the injury site was identified and parts of the connective tissue covering the spinal cord above and below the injury site were carefully dissected. Special care was taken not to damage spinal cord tissue and connective tissue directly above the injury site. To simplify the initial penetration of the thin glass micropipette into the chronically injured spinal cord, a small opening in the meninges was made using 27G needle. For transplantation, neural crest cells were detached by accutase treatment and suspended at a concentration of 100,000 cells per µl in N2 medium. The cells were then transferred into a siliconized glass micropipette (100 µm O.D.) attached to a 5 µl Hamilton syringe. The cell suspension was slowly pressure injected into the spinal cord (1.5 µl over 10 to 12 min) at a depth of 1 mm and positions approximately 1 mm rostral and caudal to the lesion site using a Stoelting’s Lab Standard Stereotaxic Instrument (Stoelting Co., Wood Dale, IL, USA). The micropipette was left in place for additional 3 min prior to being gently removed. The injury and transplantation sites were subsequently covered with fibrin glue (Tisseel Baxter AG, Zürich, Switzerland) and a thin layer of Spongostan (Johnson & Johnson Medical Ltd, Wokingham, UK) soaked in normal saline. The muscles and skin were then closed in successive layers and the transplanted animals were administered fluixin (Finadyne, Schering-Plough, Ballerup, Denmark; 2.5 mg/kg, subcutaneous), normal saline (5 ml, subcutaneous), and benzylpenicillin (Boehringer Ingelheim Animal Health Nordics A/S, Copenhagen, Denmark; 30 mg, intramuscular). All experimental animals with SCI were treated with cyclosporine A (CsA, Novartis Sverige AB, Kista, Sweden; 15 mg/kg subcutaneous) for 3 weeks after cell transplantation or medium injection. The duration of CsA treatment was based on our previous studies demonstrating markedly reduced expression of neurotrophic factors by the transplanted human stem cells at 3 weeks postoperatively^24^ and unexpected significant additional neuroprotective effects of CsA following long-term administration^25^.

### Tissue Processing

Animals were sacrificed at 3- and 16-week post-transplantation by an intraperitoneal overdose of sodium pentobarbital. For quantitative reverse transcriptase-polymerase chain reaction (qRT-PCR) and enzyme linked immunosorbent assay (ELISA), C3 and C4 spinal cord segments (rostral and caudal to the lesion site, respectively) were removed and divided sagittally prior to freezing in liquid nitrogen. For immunohistochemical analysis, animals were transcardially perfused with Tyrode’s solution followed by 4% (w/v) paraformaldehyde (PFA) in 0.1 M phosphate buffer (pH 7.4). Spinal cord segments (C2, C3–C5, and C6) were then removed and transferred into 4% (w/v) PFA in 0.1 M phosphate buffer (pH 7.4) at 4°C for 4 h. The tissue blocks were then cryoprotected in 10% (w/v) and 20% (w/v) sucrose in 0.1 M phosphate buffer (pH 7.4) for up to 5 days prior to being frozen in liquid isopentane and cryosectioned. Serial horizontal or transverse cryosections (16 µm) were dried overnight at room temperature prior to being stored at −85°C.

### Quantitative Reverse Transcriptase-Polymerase Chain Reaction

Total RNA was isolated from hESCs, neural crest cells, and spinal cord segments using the RNeasy Mini Kit (Qiagen AB, Sollentuna, Sweden) and reverse transcribed using the iScript cDNA Synthesis Kit (Bio-Rad Laboratories AB, Solna, Sweden). The resulting templates were analyzed by qPCR using a CFX96 Real Time System (Bio-Rad Laboratories AB, Solna, Sweden) and the SsoAdvanced Universal SYBR-Green Supermix (Bio-Rad Laboratories AB, Solna, Sweden). Species-specific oligonucleotides were employed in this study. These were designed by BLAST alignment of the requisite human and rat gene sequences to identify nonhomologous regions and species-specific primer pairs were designed against domains that contained the greatest base pair mismatches. The specificity of each primer pair was verified by RT-PCR using total RNA isolated from neural crest cells and rat C4 spinal cord segments using a T100 Thermal Cycler (Bio-Rad Laboratories AB, Solna, Sweden) and the OneStep RT-PCR kit (Qiagen AB, Sollentuna, Sweden). Details of all oligonucleotide sequences used in this study are given in Supplemental Table S2.

### Immunostaining

Neural crest cells were fixed in ice-cold 4% (w/v) PFA in phosphate buffered saline (PBS) for 10 min and immunostaining as described previously^20,26^. The cells were washed three times for 5 min with PBST [PBS and 0.1% (v/v) Triton X-100], blocked for 1 h at room temperature in 10% (v/v) fetal calf serum (FCS) in PBST and then incubated overnight at 4°C with the required primary antibodies diluted in 10% (v/v) FCS in PBST. Cells were subsequently washed three times for 5 min with PBST and then incubated for 1 h at room temperature with 5 ng/ml 4’,6-diamidino-2-phenylindole (DAPI) (Sigma-Aldrich, St. Louis, MO, USA) and the required secondary antibodies (Thermo Fisher Scientific, Stockholm, Sweden, 1:500) diluted in 10% (v/v) FCS in PBST. Cells were finally washed three times for 5 min in PBST and mounted using Vectashield Mounting Medium (Vector Laboratories, Inc., Burlingame, CA, USA). Spinal cord cryosections were rehydrated two times for 30 min in PBSAB [PBS containing 0.1% (w/v) sodium azide and 0.1% (w/v) bovine serum albumin]. The slides were then incubated for 1 h in blocking solution [5% (v/v) normal horse serum, 5% (v/v) normal goat serum, and 0.2% (v/v) Triton-X100] in PBSAB prior to being incubated for 2 h with the required primary antibodies diluted in PBSAB. The slides were then washed with PBSAB and subsequently incubated for 1 h with the required secondary antibodies (Thermo Fisher Scientific, Stockholm, Sweden, 1:500) diluted in blocking solution. The slides were finally washed three times for 5 min in PBS and mounted using ProLong antifade mounting medium with DAPI (Thermo Fisher Scientific, Stockholm, Sweden). All incubations were performed at room temperature. Antibody specificity was verified by omission of primary antibodies. Details of all primary antibodies used in this study are provided in Supplemental Table S3.

### Conditioned Medium

Neural crest cells were plated onto Matrigel-coated flasks (50,000 per cm^2^) and cultured in N2 medium supplemented with 10 ng/ml bFGF and 20 ng/ml EGF. Dispersed hESCs were seeded as monolayers on Matrigel-coated culture vessels (50,000 per cm^2^)^23^ and grown in hESC medium containing 10 ng/ml bFGF and 20 ng/ml EGF. Conditioned medium was harvested from all cultures after 48 h incubation and filtered using a Steriflip Filtration System (Merck Chemicals and Life Science AB, Solna, Sweden).

### Enzyme Linked Immunosorbent Assay

Spinal cord segments were homogenized in ice-cold RIPA Buffer (Thermo Fisher Scientific, Stockholm, Sweden) containing Protease Inhibitor Cocktail (Sigma-Aldrich, St. Louis, MO, USA) using a TissueRuptor (Qiagen AB, Sollentuna, Sweden). The homogenate was agitated on an orbital shaker at 4°C for 2 h prior to the insoluble material being pelleted by centrifugation at 13,000 × *g* for 10 min at 4°C. The supernatant was carefully removed and snap-frozen in liquid nitrogen. Soluble protein concentrations were determined using the DC Protein Assay (Bio-Rad Laboratories AB, Solna, Sweden). ELISAs were performed on C3 spinal cord protein extracts and conditioned media using RayBio Sandwich ELISA kits (RayBiotech Life, Inc., Peachtree Corners, GA, USA).

### Neurite Outgrowth Assay

Primary sensory neurons were dissociated from the DRG of adult female Sprague-Dawley rats (*n* = 6) as described previously^24^. The isolated neurons were seeded on 20 µg/ml laminin coated culture vessels and grown for 24 h in Neurobasal-A supplemented with B27, 0.5 mM l-glutamine, and 1% (v/v) PeSt. The primary cultures were then briefly washed in PBS prior to the addition of conditioned media (0.5 ml). The cultures were then incubated for a further 72 h. Total neurite outgrowth analyses were performed as described previously^20^.

### Image Analysis and Quantification

Images were captured using a Zeiss LSM 710 confocal or Nikon Eclipse E80i microscope coupled to a Nikon DS-U2 digital camera. All images were analyzed and compiled using Fiji^27^, Adobe Photoshop, Adobe Illustrator (Adobe, San Jose, CA, USA), and Image-Pro Plus (Media Cybernetics, Inc., Rockville, MD, USA). Density of the descending motor 5-hydroxytryptamine (5-HT^+^) raphespinal axons was quantified in the trauma zone and in the Rexed lamina IX of the C2 and C6 ventral horns (Fig. 4A, B). Aberrant ingrowth of the calcitonin gene-related peptide (CGRP^+^) sensory axons into Rexed lamina III was studied in the C2 dorsal horn (Fig. 5A, B), and the reaction of the GFAP^+^ astrocytes and OX42^+^ microglia was evaluated at distances of 2,000 to 2,250 µm rostral and caudal from the injury site at the level of the central canal in Rexed lamina VII. In addition, the density of GFAP^+^ astrocytes was analyzed in the scar tissue surrounding injury site. All quantifications were performed on cojoining regions of interest, regions of interest (2,500 µm^2^) that were randomly placed on each image prior to analysis using custom macros written in Adobe Photoshop.

### Vertical Cylinder Test

Spontaneous forelimb use was assessed using vertical exploration of a clear plastic cylinder^28^. All experimental animals were recorded for 5 min upon placement within the cylinder and the extent of motor recovery of the forelimb was assessed from these video recordings. The following parameters were scored: (i) independent or simultaneous use of the injured and uninjured forelimb for initial wall contact during a full rear, (ii) independent or simultaneous use of the injured and uninjured forelimb for wall exploration following a full rear, and (iii) independent or simultaneous use of the injured and uninjured forelimb for landing following exploration. These counts were then employed to calculate the percentage of independent injured, independent uninjured, or simultaneous forelimb use for rearing, exploration, and landing, respectively. Testing was performed at 8-, 12-, and 16-week post-transplantation.

### Statistical Analyses

Statistical analyses were performed using GraphPad Prism 7 (GraphPad Software, San Diego, CA, USA). Unpaired two-tailed Student’s *t*-tests, one-way analysis of variance (ANOVA) followed by Tukey’s multiple comparisons test or a repeated measures two-way ANOVA followed by Sidak’s multiple comparisons test were used to determine all statistical significances. Data on all graphs are presented as the mean and the standard error of the mean. *P*-values are indicated as follows: ns, not significant, *P ≤ 0.05, **P ≤ 0.01, ***P ≤ 0.001, and ****P ≤ 0.0001.

## Results

### Differentiation and Characterization of hESC-derived Neural Crest Cells

**Figure 1.**
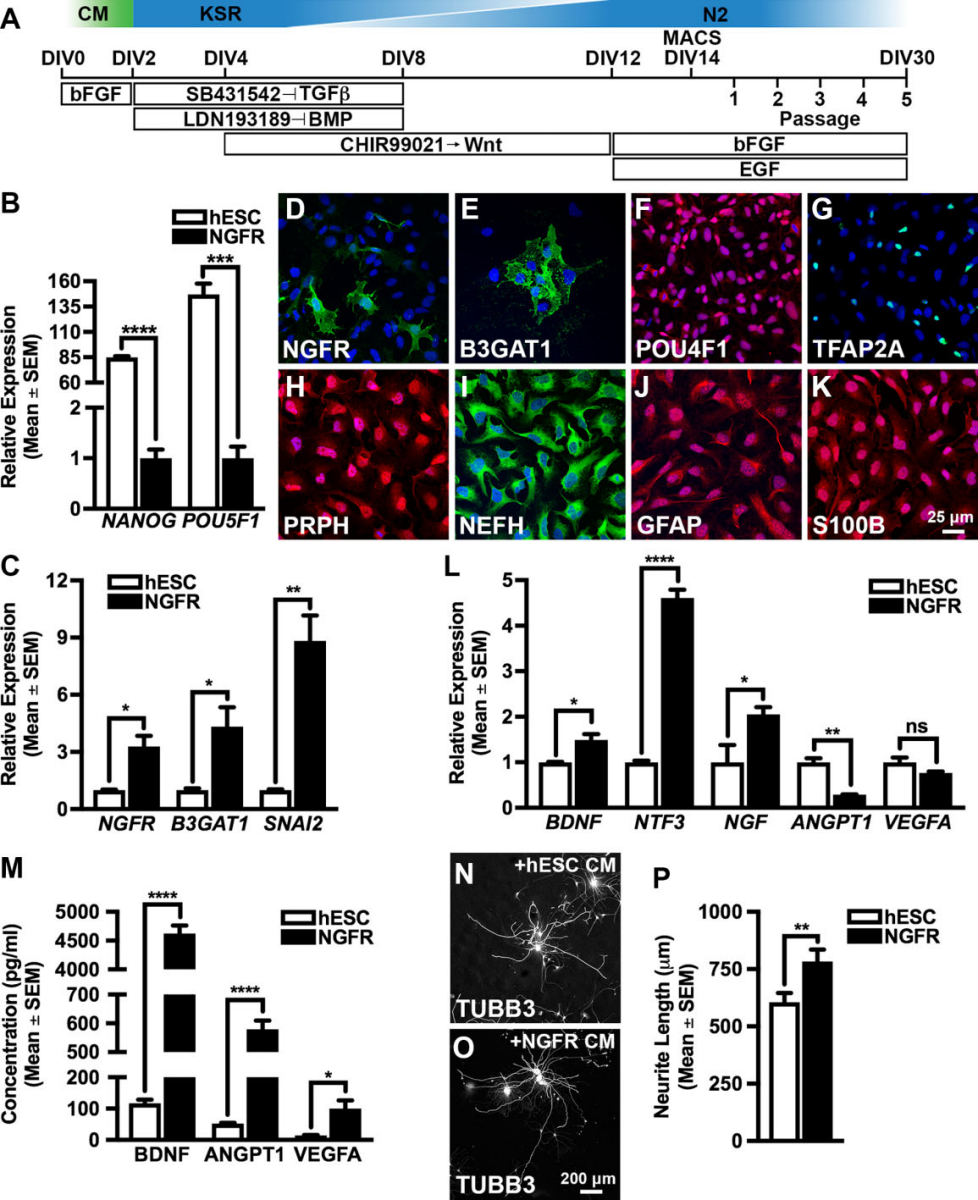
Differentiation and characterization of hESC-derived neural crest cells. (A) Schematic diagram outlining the neural crest differentiation protocol used in this study. (B) qRT-PCR analysis of pluripotent markers in the parental hESC culture (DIV0) and the enriched NGFR^+^ population (DIV14). Relative expression levels are given with respect to the NGFR^+^ population whose value has been set to 1. (C) qRT-PCR analysis of neural crest cell markers in the parental hESC culture (DIV0) and the enriched NGFR^+^ population (DIV14). Relative expression levels are given with respect to the hESC population whose value has been set to 1. (D to K) The NGFR^+^ cell population (DIV30) express established neural crest (B3GAT1, POU4F1, and TFAP2A) in addition to pan-neuronal (PRPH and NEFH) and glial (GFAP and S100B) markers, respectively. (L) qRT-PCR analysis of neurotrophic (*BDNF*, *NT-3*, and *NGF*) and angiogenic (*ANGPT1* and *VEGFA*) markers in the parental hESC culture (DIV0) and NGFR^+^ enriched cells (DIV30). Relative expression levels are given with respect to the hESC population whose value has been set to 1. (M) ELISA analysis of conditioned medium harvested from the original hESC culture (DIV2) and NGFR^+^ enriched cells (DIV30). (N to P) Quantitative total neurite outgrowth analysis of rat primary DRG neurons exposed to conditioned medium harvested from NGFR^+^ enriched cells (DIV30) or hESCs (DIV2). All data represent the mean ± SEM. Statistical significances were calculated using unpaired two-tailed Student’s *t*-tests using data derived from three independent differentiation experiments. *P*-values are denoted as follows: *P ≤ 0.05, **P ≤ 0.01, ***P ≤ 0.001, and ****P ≤ 0.0001. Scale bars: (D to K) 25 µm and (N, O) 200 µm. ANGPT1: angiopoietin-1; BDNF: brain-derived neurotrophic factor; CM: conditioned medium; DIV: days *in vitro*; ELISA: enzyme linked immunosorbent assay; GFAP: glial fibrillary acidic protein; hESC: human embryonic stem cell; KSR: KnockOut Serum Replacement medium; MACS: magnetic activated cell sorting; NGF: nerve growth factor; NT-3: neurotrophin-3; qRT-PCR: quantitative reverse transcriptase-polymerase chain reaction; SEM: standard error of the mean; S100B: S100 calcium-binding protein B; VEGFA: vascular endothelial growth factor A.

hESCs were differentiated toward neural lineages by small molecule inhibition of SMAD and WNT-signaling pathways^23^ and the NGFR^+^ (nerve growth factor receptor) cell population was enriched by MACS at 14 days *in vitro* (DIV14). The isolated cells were then expanded for a maximum of five passages in the presence of bFGF/EGF (DIV14 to DIV30) (Fig. 1A). We first examined changes in gene expression during the differentiation protocol by comparing the transcription level of key marker genes between the parental hESC (DIV0) and the NGFR^+^ cell populations (DIV14 or DIV30) (Fig. 1B, C). A downregulation of pluripotent genes (*NANOG* and *POU5F1*, also known as *OCT-4*)^29^ (Fig. 1B) and parallel upregulation of neural crest cell markers (*NGFR*, *B3GAT1*, and *SNAI2*)^30^ (Fig. 1C) was observed in the NGFR^+^ cell population (DIV14) compared to the hESC cultures (DIV0). Furthermore, subsets of the NGFR^+^ population (DIV30) (22 ± 3%, *n* = 67/308) (Fig. 1D) expressed the neural crest markers B3GAT1 (beta-1,3-glucuronyltransferase 1) (26 ± 4%, *n* = 87/333), POU4F1 (POU domain, class 4, transcription factor 1) (88 ± 3%, *n* = 287/325), and TFAP2A (transcription factor AP-2 alpha) (22 ± 1%, *n* = 82/369)^17^ (Fig. 1E–G). Taken together, the marker signature analyses demonstrated a reduction in pluripotency and a corresponding induction of the neural crest lineage in the NGFR^+^ population. Moreover, the NGFR^+^ cells (DIV30) exhibited multipotent characteristics as demonstrated by the robust expression of the intermediate neurofilaments PRPH (peripherin) (94 ± 1%, *n* = 127/135) and NEFH (neurofilament heavy polypeptide) (95 ± 2%, *n* = 130/137) (Fig. 1H, I) in addition to the glial cell markers GFAP (glial fibrillary acidic protein) (91 ± 4%, *n* = 107/117) and S100B (S100 calcium-binding protein B) (90 ± 2%, *n* = 143/159) (Fig. 1J, K)^31,32^.

We next compared the trophic factor expression profile of the enriched NGFR^+^ population (DIV30) with that of the parental hESCs (DIV0 and DIV2) by qRT-PCR and ELISA (Fig. 1L, M). An increase in neurotrophic gene transcription (*BDNF*, *NT-3*, and *NGF*) but reduced levels of angiogenic factor expression (*ANGPT1* and *VEGFA*) was observed in the NGFR^+^ cells (DIV30) relative to the hESCs (DIV0) (Fig. 1L). We also observed trophic factor secretion (BDNF, ANGPT1 and VEGFA) in conditioned medium harvested from the NGFR^+^ cells (DIV30) but not in the hESC population (DIV2) (Fig. 1M). *In vitro* neurite outgrowth assays were therefore performed to assess the bioactivity of these secreted trophic factors. Exposure of rat primary DRG neurons to NGFR^+^ conditioned medium (DIV30) induced the elaboration of TUBB3^+^ (tubulin beta class 3) neurites that were consistently longer than the corresponding neurite outgrowth of primary neuronal cultures exposed to hESC-conditioned medium (DIV2) (Fig. 1N–P).

### Transplantation of Neural Crest Cells into Rat Cervical SCI Model

**Figure 2.**
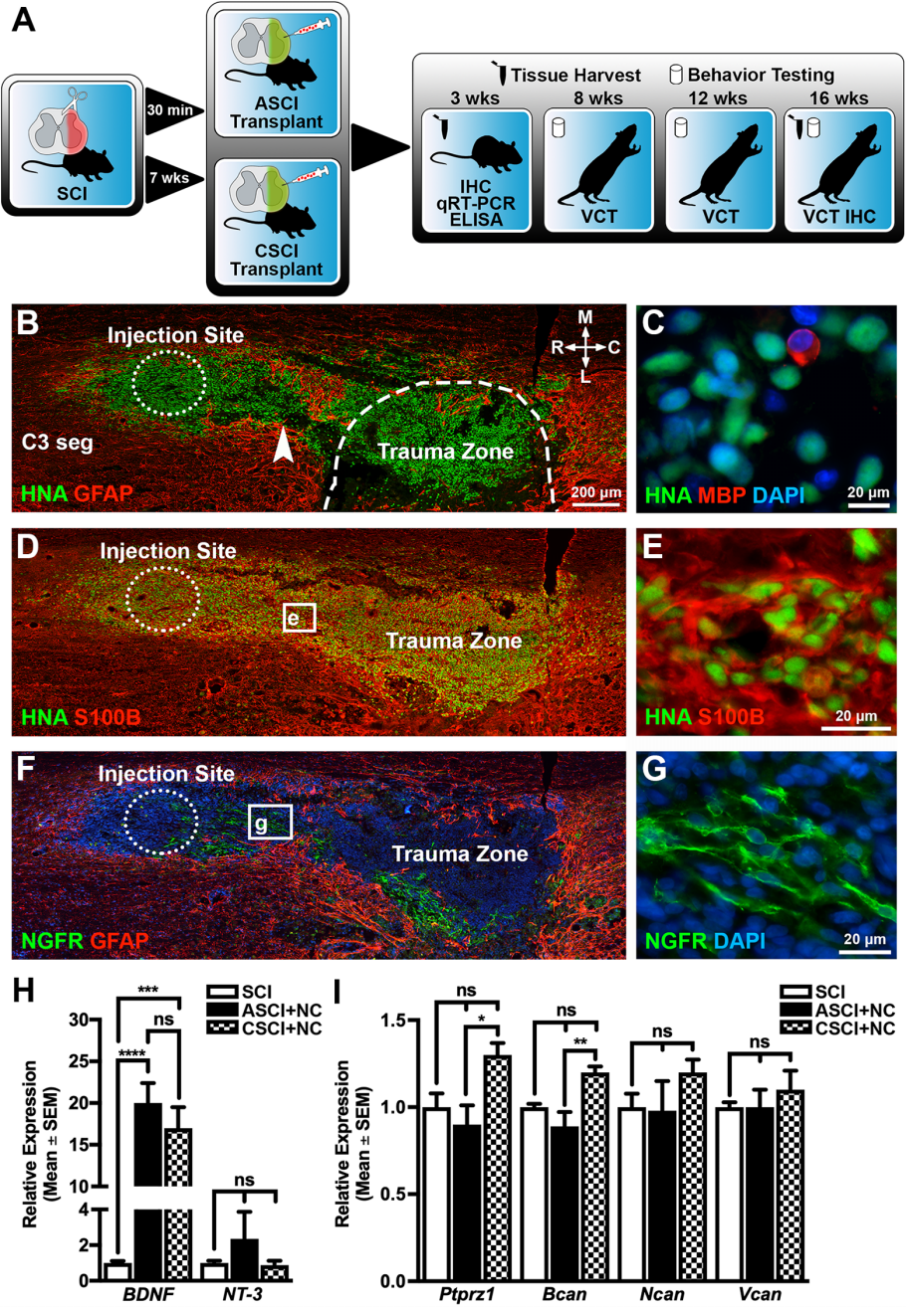
Transplantation of differentiated neural crest cells into a rat cervical spinal cord injury model. (A) Schematic diagram detailing the experimental approach and timeline used in this study. (B to G) Representative serial horizontal C3–C4 spinal cord sections demonstrating HNA^+^ neural crest cells 3 weeks after transplantation following ASCI. Some HNA^+^ neural crest cells differentiate along glial cell lineages and express S100B and NGFR^+^, but are negative for MBP (C). Host GFAP^+^ astrocytes show very limited migration into the transplantation sites. Images in (C, F, and G) are counterstained with DAPI. Dotted circles indicate the injection sites and the dashed line indicates the trauma zone border. Arrowhead shows pressure-injected neural crest cells forming a tract connecting injection site with trauma zone. (H) qRT-PCR analysis of C4 spinal cord segments using human-specific primers demonstrates that the neural crest cells express elevated levels of *BDNF* following transplantation in both ASCI and CSCI animals relative to SCI controls 3 weeks after transplantation. (I) qRT-PCR analysis of C4 spinal cord segments using rat-specific primers demonstrates that host cells express elevated levels of *Ptprz1* and *Bcan* in CSCI animals relative to the ASCI group 3 weeks after transplantation. Relative expression levels for all qRT-PCR experiments are given with respect to the SCI control group whose value has been set to 1. All data represent the mean ± SEM. Statistical significances were calculated using a one-way ANOVA followed by Tukey’s multiple comparisons test using data derived from ASCI +NC (*n* = 5), transplanted CSCI +NC (*n* = 4), and ASCI control (*n* = 5) groups. *P* values are indicated as follows: *P ≤ 0.05, **P ≤ 0.01, ***P ≤ 0.001, and ****P ≤ 0.0001. Scale bars: (B, C, and E) 200 µm, (D) 20 µm, and (F) 20 µm. ANOVA: analysis of variance; ASCI +NC: cell transplantation into acute spinal cord injury; Bcan: brevican; BDNF: brain-derived neurotrophic factor; C: caudal; C3 seg: cervical spinal segment 3; CSCI +NC: cell transplantation into chronic spinal cord injury; DAPI: 4′,6-diamidino-2-phenylindole; GFAP: glial fibrillary acidic protein; HNA: human nuclear antigen; IHC: immunohistochemistry; L: lateral; M: medial; MBP: myelin basic protein; Ncan: neurocan; NT-3: neurotrophin-3; Ptprz1: phosphacan; qRT-PCR: quantitative reverse transcriptase-polymerase chain reaction; R: rostral; S100B: S100 calcium-binding protein B; SCI: spinal cord injury control; SEM: standard error of the mean; Vcan: versican; VCT: vertical cylinder test.

Our *in vitro* experiments demonstrated that multipotent neural crest cells could be rapidly differentiated from hESCs and that the enriched cells secreted biologically active trophic factors that stimulated rat primary DRG neuron outgrowth. These features suggested that the neural crest cells could be viable cell-based therapy candidates. We therefore assessed their *in vivo* regenerative potential in a rat cervical SCI model (Fig. 2A). We observed numerous HNA^+^ (human nuclear antigen) neural crest cells in both ASCI and CSCI 3 weeks after transplantation (Fig. 2B–E; Supplemental Fig. 1). In ASCI we found that pressure-injected cells displaced host GFAP^+^ astrocytes and formed a narrow tract that connected the injection site with the trauma zone (Fig. 2B, arrowhead). In contrast, transplantation into CSCI did not induce any significant cell dispersion from the injection site (Supplemental Fig. S1). Staining for myelin basic protein (MBP) did not reveal any double-labeled HNA^+^/MBP^+^ transplanted cells, although we found singe HNA^−^ and MBP^+^ cells among HNA^+^ neural crest cells in the trauma zone (Fig. 2C). Numerous HNA^+^ neural crest cells differentiated along glial cell lineages within the spinal cord environment as demonstrated by the robust coexpression of S100B (Fig. 2D, E). Using human-specific antibodies we also found separate groups of NGFR^+^ cells in the injection sites and trauma zone (Fig. 2F, G). The lower number of NGFR^+^ cells in the transplanted animals could be accounted for by several possible mechanisms including downregulation of receptor expression following stress caused by transplantation and/or the hostile environment of the reactive nerve tissue.

We did not observe any NeuN^+^ cells in the injection sites 3 weeks postoperatively and we did not find any HNA^+^ cells at 16 weeks after transplantation. Since neural crest cells can potentially differentiate into non-neuronal cells, we performed additional immunostaining with antibodies against epithelial markers (cytokeratin), mesenchymal cells/fibroblasts (human-specific Thy-1/CD90), adipocytes (Glut4), and alpha-smooth muscle actin. We did not find any cells stained for these additional neural crest lineage markers. Interestingly, qRT-PCR analysis using a panel of human-specific primers demonstrated that the neural crest cells continued to express elevated levels of *BDNF* transcripts in C4 spinal cord segments in both ASCI and CSCI animals for up to 3 weeks following transplantation (Fig. 2H). In addition, while detectable levels of *NT-3* mRNA were also observed in both groups, the normalized values were similar to SCI controls (Fig. 2H). However, all other qRT-PCR analysis of human-specific genes (*NGF*, *ANGPT1*, and *VEGFA*) and ELISAs for trophic molecules (BDNF, ANGPT1, and VEGFA) that were detected during the *in vitro* characterization of the neural crest cells were undetectable in C4 spinal cord segments. Furthermore, qRT-PCR analyses demonstrated that cell transplantation did not affect the expression of *Ptprz1*, *Bcan*, *Ncan*, and *Vcan* chondroitin sulphate proteoglycan genes when compared to SCI alone (Fig. 2I). Notwithstanding, the differences observed in the expression of *Ptprz1* and *Bcan* between the ASCI and CSCI could reflect the differences in the timing of cell transplantation.

### Effects of Neural Crest Cell Transplantation on Axon Sprouting and Glial Cell Reaction

**Figure 3.**
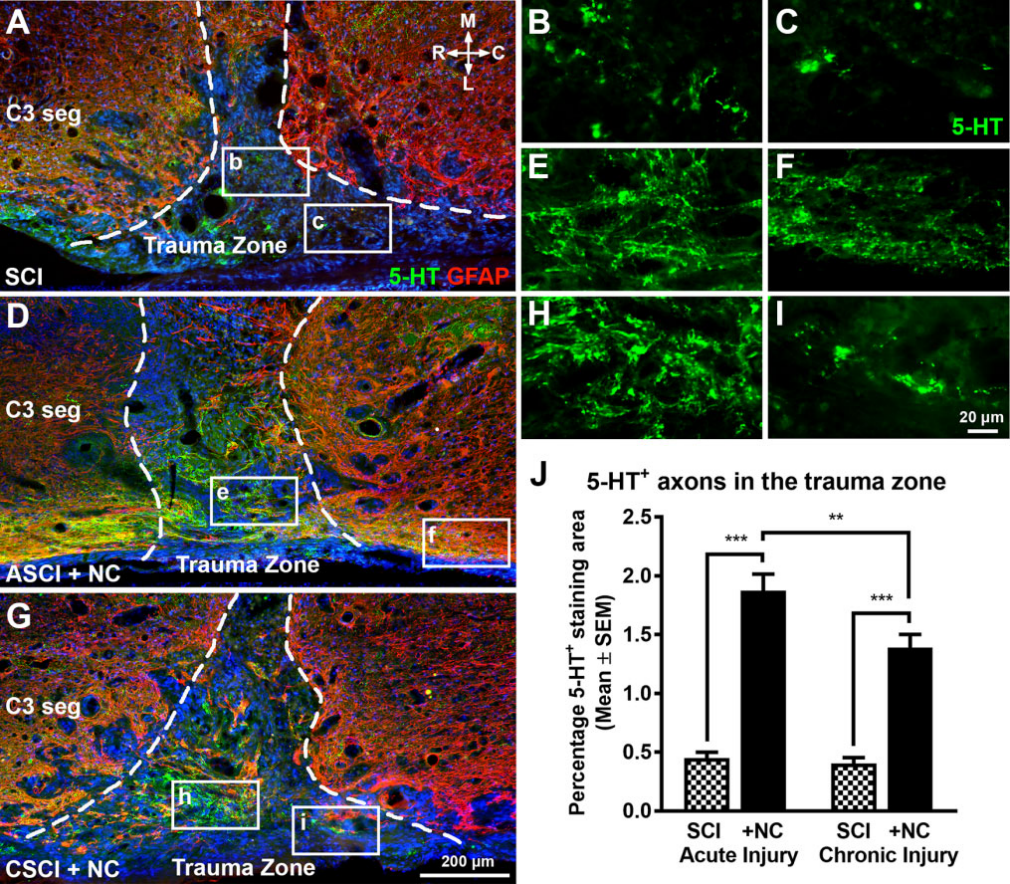
The effects of neural crest cell transplantation on regeneration of 5-HT^+^ raphespinal axons. (A to C) Representative horizontal spinal cord sections demonstrate that control animals have single 5-HT^+^ raphespinal terminals within the injury site 16 weeks post-operatively. (D to I). In contrast, an increase in 5-HT^+^ ingrowth into the trauma zone of both ASCI (D and E) and CSCI (G and H) animals is observed following neural crest cell transplantation. Images are counterstained with DAPI to demonstrate the non-glial part of the scar. (C and D) Dashed lines indicate borders of the trauma zone. (J) Histogram showing the regenerating 5-HT^+^ terminal density in the trauma zone. Transplantation of neural crest cells stimulated axonal growth in both acute and chronic injury groups. All data represent the mean ± SEM. Statistical significances were calculated using a one-way ANOVA followed by Tukey’s multiple comparisons test using data derived from ASCI (*n* = 5), ASCI +NC (*n* = 7), CSCI (*n* = 5), and CSCI +NC (*n* = 5) groups. *P*-values are denoted as follows: **P ≤ 0.01 and ***P ≤ 0.001. Scale bars: (A, D, and G) 200 µm and (B, C, E, F, H, and I) 20 µm. 5-HT^+^: 5-hydroxytryptamine; ANOVA: analysis of variance; ASCI: acute spinal cord injury; ASCI +NC: cell transplantation into ASCI; C: caudal; C3 seg: cervical spinal cord segment 3; CSCI: chronic spinal cord injury; CSCI +NC: cell transplantation into CSCI; DAPI: 4′,6-diamidino-2-phenylindole; L: lateral; M: medial; NC: neural crest cells; R: rostral; SCI: spinal cord injury control; SEM: standard error of the mean.

**Figure 4.**
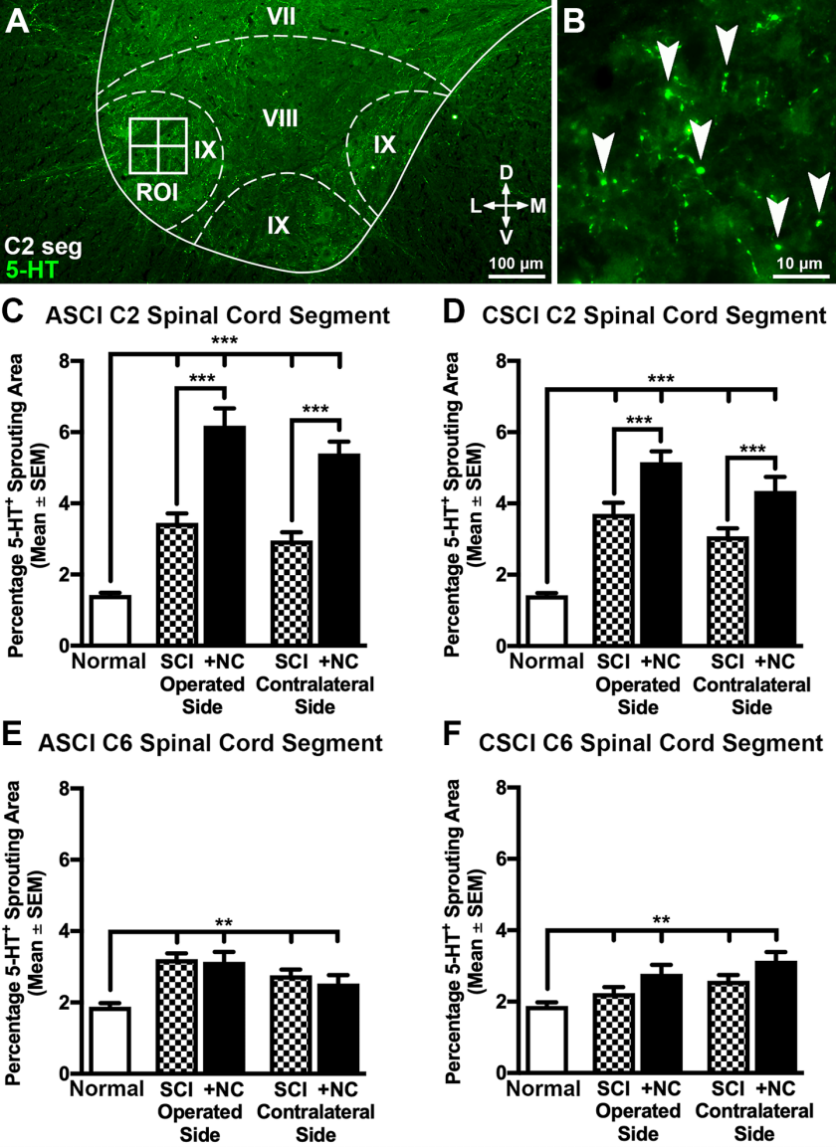
The effects of neural crest cell transplantation on 5-HT^+^ raphespinal axon sprouting rostral and caudal to the injury site. (A and B) Representative transverse spinal cord sections showing the strategy employed to quantify the density of 5-HT^+^ raphespinal terminals 16 weeks postoperatively. The ventral horn was defined and four cojoining ROIs (2,500 µm^2^) were superimposed laterally on Rexed lamina IX using custom macros (A). The area of serotoninergic 5-HT^+^ axon sprouting was subsequently quantified manually within each ROI (B, arrowheads). (C and D) Histograms showing the density of 5-HT^+^ terminals in rostral C2 spinal cord segments. Cervical SCI alone caused an increase in the number of raphespinal terminals on both the operated and contralateral sides relative to the control group. Transplantation of neural crest cells further stimulated this regenerative response in both ASCI (C) and CSCI (D) groups. (E and F) Histograms showing the density of 5-HT^+^ terminals in caudal C6 spinal cord segments. Cervical SCI alone caused an increase in the density of raphespinal terminals on both the operated and contralateral sides relative to the control group. However, transplantation of neural crest cells did not increase 5-HT^+^ densities in both ASCI and CSCI treatment groups. All data represent the mean ± SEM. Statistical significances were calculated using a one-way ANOVA followed by Tukey’s multiple comparisons test using data derived from normal uninjured control (*n* = 5), ASCI (*n* = 5), ASCI +NC (*n* = 7), CSCI (*n* = 5), and CSCI +NC (*n* = 5) groups. *P*-values are denoted as follows: **P ≤ 0.01 and ***P ≤ 0.001. Scale bars: (a) 100 µm and (b) 10 µm. 5-HT^+^: 5-hydroxytryptamine; ANOVA: analysis of variance; ASCI: acute spinal cord injury; C2: cervical vertebrae 2; C2 seg: cervical spinal cord segment 2; C6: cervical spinal cord segment 6; CSCI: chronic spinal cord injury; D: dorsal; L: lateral; M: medial; +NC: spinal cord injury plus neural crest cell transplantation; Normal: uninjured control; ROI: region of interest; SCI: spinal cord injury only; SEM: standard error of the mean; V: ventral.

We observed that neural crest expressed elevated levels of *BDNF* for up to 3 weeks post-transplantation (Fig. 2H). These results prompted us to assess whether the transplanted cells had any long-term effects on regeneration and sprouting of descending motor raphespinal (5-HT^+^) and aberrant sprouting of sensory (CGRP^+^) axons, respectively. Sixteen weeks after SCI we found single 5-HT^+^ raphespinal axons present within the injury site of control animals (Fig. 3A–C). In contrast, cell transplantation induced a four-fold increase in the density of 5-HT^+^ axons in animals with ASCI (Fig. 3D–F and J) and a three-fold increase in the density of regenerating axons in animals with CSCI (Fig. 3G–I and J) when compared with time-matched SCI controls, respectively. We subsequently quantified 5HT^+^ sprouting in rostral C2 and caudal C6 cervical segments (Fig. 4A, B and Supplemental Fig. S2). SCI alone caused an increase in the number of raphespinal axon arborizations in both the operated and contralateral ventral horn of all animals analyzed relative to the uninjured control group (Fig. 4C–F). Transplantation further stimulated this bilateral regenerative response rostral to the trauma zone in C2 spinal cord segments since an increase in the area occupied by 5-HT^+^ terminals was observed in both ASCI (Fig. 4C) and CSCI (Fig. 4D) animals. In contrast, transplantation had no effect on the density of 5-HT^+^ terminals in C6 segments caudal to the lesion site (Fig. 4E, F).

**Figure 5.**
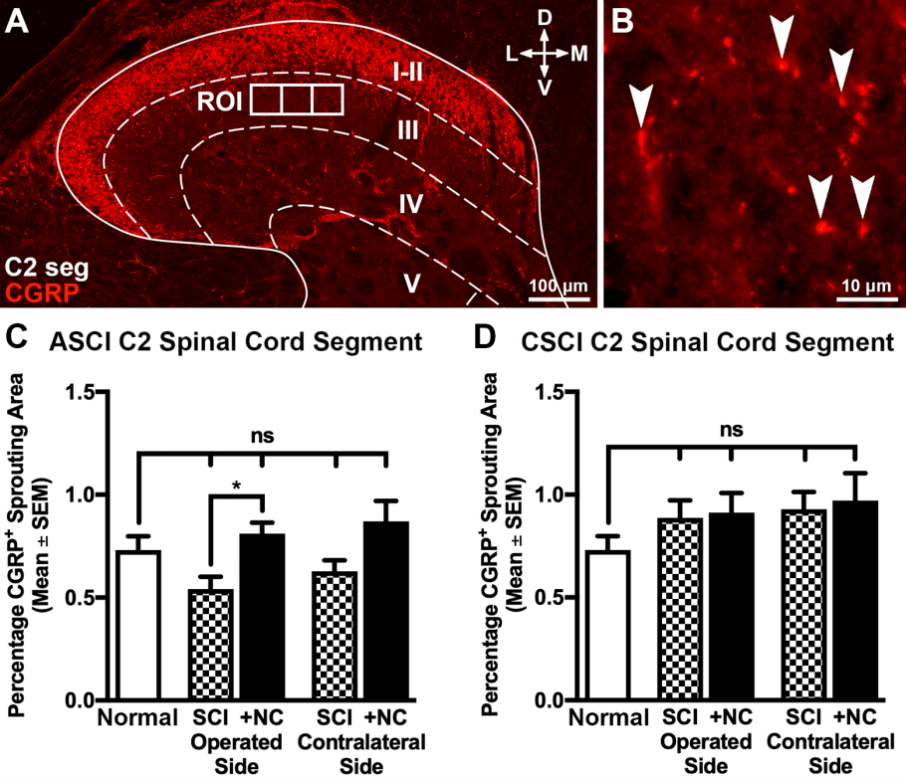
The effects of neural crest cell transplantation on aberrant sprouting of CGRP^+^ sensory axons. (A and B) Transverse spinal cord section showing the strategy employed to quantify the density of CGRP^+^ axons 16 weeks postoperatively. The dorsal horn was defined and three cojoining ROIs (2,500 µm^2^) were superimposed on Rexed lamina III using custom macros (A). The area occupied by CGRP^+^ axons within each ROI was subsequently quantified (B, arrowheads). (C and D) Histograms showing the density of CGRP^+^ axons in Rexed lamina III of rostral C2 dorsal horn segments. No differences were observed between control and treatment groups (C and D). All data represent the mean ± SEM. Statistical significances were calculated using a one-way ANOVA followed by Tukey’s multiple comparisons test using data derived from normal uninjured control (*n* = 5), ASCI (*n* = 5), ASCI +NC (*n* = 7), CSCI (*n* = 5), and CSCI +NC (*n* = 5) groups. *P*-values are denoted as follows: *P ≤ 0.05. Scale bars: (a) 100 µm and (b) 10 µm. ANOVA: analysis of variance; ASCI: acute spinal cord injury; C2 seg: cervical spinal cord segment 2; CSCI: chronic spinal cord injury; CGRP^+^: calcitonin gene-related peptide; D: dorsal; L: lateral; M: medial; +NC: spinal cord injury plus neural crest cell transplantation; Normal: uninjured control; ROI: region of interest; SCI: spinal cord injury only; SEM: standard error of the mean; V: ventral.

**Figure 6.**
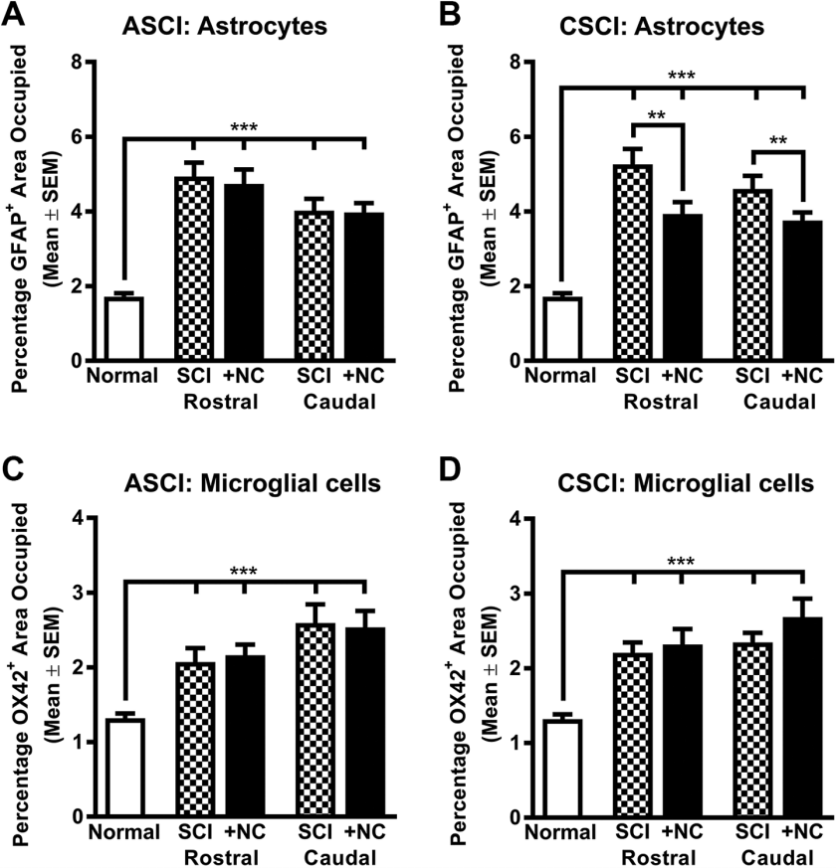
The effects of neural crest cell transplantation on glial cell reactions following spinal cord injury. (A and B) Histograms showing the area occupied by GFAP^+^ astrocytes in normal uninjured animals (Normal), animals at 16 to 23 weeks after spinal cord injury alone (ASCI, CSCI) and at 16 weeks following neural crest cell transplantation (ASCI +NC, CSCI +NC) both rostral and caudal to the injury site (2,000 to 2,250 µm). Cervical SCI alone caused an increase in the area occupied by GFAP^+^ astrocytes both rostral and caudal to the injury site. Transplantation of neural crest cells did not attenuate this gliogenic response in the ASCI group (A) but decreased the area occupied by GFAP^+^ astrocytes in CSCI animals (B). (C and D) Histograms showing the density of OX42^+^ microglial cells. Cervical SCI alone caused an increase in the area occupied by microglial cells both rostral and caudal to the injury site. No improvement in area occupied by OX42^+^ cells was observed in either the ASCI (C) or CSCI (D) treatment groups following neural crest cell transplantation. All data represent the mean ± SEM. Statistical significances were calculated using a one-way ANOVA followed by Tukey’s multiple comparisons test using data derived from normal uninjured control (*n* = 5), ASCI (*n* = 5), ASCI +NC (*n* = 7), CSCI (*n* = 5), and CSCI +NC (*n* = 5) groups. *P*-values are denoted as follows: **P ≤ 0.01, ***P ≤ 0.001. ANOVA: analysis of variance; ASCI: acute spinal cord injury; CSCI: chronic spinal cord injury; GFAP: glial fibrillary acidic protein; +NC: spinal cord injury plus neural crest cell transplantation; Normal: uninjured control; SCI: spinal cord injury only; SEM: standard error of the mean.

We also assessed the long-term effects of cell transplantation on aberrant sensory (CGRP^+^) axon sprouting in lamina III of the C2 cervical dorsal horn (Fig. 5A, B and Supplemental Fig. S2). We observed that SCI alone and SCI followed by transplantation of neural crest cells had no effects on the density of CGRP^+^ terminals in both the operated and contralateral C2 cervical dorsal horn of ASCI and CSCI animals (Fig. 5C, D). SCI also induced the activation of both GFAP^+^ astrocytes and OX42^+^ (integrin alpha-M) microglial cells both rostral and caudal to the lesion and injection sites (Fig. 6A–D, Supplemental Fig. S2A). As expected, an increase in the density of GFAP^+^ astrocytes was also found in the scar tissue surrounding injury site (Supplemental Fig. S3B, C). However, the neural crest cells had no effect on astrocytic activation in the scar region but reduced astrocytic reactivity rostral and caudal to the injury site when transplanted in CSCI animals (Fig. 6B and Supplemental Fig. S3). No effects on microglial cell activation was observed in both treatment groups (Fig. 6C, D). Taken together, our data demonstrate that transplantation of neural crest cells promoted raphespinal axon ingrowth into the trauma zone in addition to sprouting rostral to injury site and did not induce aberrant sprouting of CGRP^+^ axons. Moreover, the transplanted cells attenuated astrocytic reactivity at some distance from the injury and injection sites following CSCI but had no effect on the astrocytes in the scar and host microglial response.

### Neural Crest Cell Transplantation Improves Locomotor Activity Following Cervical SCI

**Figure 7.**
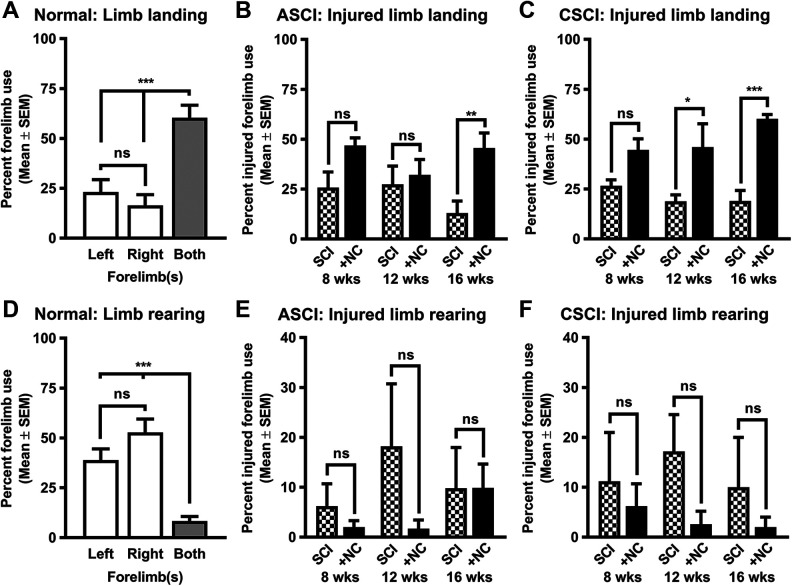
Neural crest cell transplantation partially improves locomotor activity following spinal cord injury. (A, D) Histograms showing the use of forelimbs by normal uninjured animals during landing and rearing. (B, C) Histograms showing that neural crest cell transplantation progressively improves the spontaneous use of the injured forelimb for landing in both (B) ASCI and (C) CSCI treatment groups compared to SCI control animals. (E, F) Histograms showing that neural crest cell transplantation does not improve injured forelimb use during rearing behavior in both (E) ASCI and (F) CSCI treatment groups relative to SCI control animals. All data represent the mean ± SEM. Statistical significances for normal uninjured control animals were calculated with a help of one-way ANOVA followed by Tukey’s multiple comparisons test using data derived from an independent control group (*n* = 10). Independently, statistical significances for spinal cord injury and cell transplantation (8, 12, and 16 weeks) were calculated using a repeated-measures two-way ANOVA followed by Sidak’s multiple comparisons test using data derived from ASCI +NC (*n* = 7), ASCI (*n* = 5), CSCI +NC (*n* = 5), and CSCI (*n* = 5) groups. *P*-values are denoted as follows: *P ≤ 0.01, **P ≤ 0.01, and ***P ≤ 0.001. ANOVA: analysis of variance; ASCI: acute spinal cord injury; CSCI: chronic spinal cord injury; +NC: spinal cord injury plus neural crest cell transplantation; Normal: uninjured control; SCI: spinal cord injury only; SEM: standard error of the mean.

The improvement of upper limb motor function is one of the fundamental milestones that must be realized if cell transplantation therapies are to be considered as a viable clinical treatment option following cervical SCI^11^. We therefore assessed spontaneous forelimb use by means of a vertical cylinder test in transplanted ASCI and CSCI groups at 8, 12, and 16 weeks after cell transplantation (Fig. 7 and Supplemental Fig. S4). These time points were chosen because motor recovery could take up to several months after SCI^33^ and the aim of this study was to establish the long-term effects of cell transplantation rather than to demonstrate possible differences in the rate of functional improvements. Moreover, this test is routinely used to assess motor recovery following cervical SCI^34^ and out of all motor activities scored we observed that neural crest cell transplantation progressively improved the spontaneous use of the injured forelimb for landing in both ASCI and CSCI groups (Fig. 7A–C). In contrast, cell transplantation did not improve injured forelimb use during rearing behavior and all groups relied extensively on the uninjured contralateral forelimb for initial cylinder contact (Fig. 7D–F). Taken together, the vertical cylinder test analyses demonstrated that transplantation of neural crest cells stimulated steady improvement of locomotor function in both ASCI and CSCI groups in a time-dependent manner.

## Discussion

The neural crest is a transient cell population that migrates out from the dorsal neural tube and differentiates into several cell lineages within the developing nervous system^35^. Despite this multipotent capability the efficacy of neural crest cells for the preclinical treatment of SCI remain largely unexplored and is due in part to their transient nature which makes their batch isolation from animal models a difficult task^11^. The work presented in this study therefore demonstrates that neural crest cells can be rapidly derived from hESCs in unlimited quantities. Moreover, the differentiated cells secreted biologically active trophic factors and were able to stimulate axon regeneration and sprouting which in turn promoted partial motor recovery in both ASCI and CSCI animal models. Our observations are in agreement with previous studies where rodent neural crest cells were used successfully in both ASCI and dorsal root avulsion models in addition to secreting BDNF that reduced neuronal loss following *in vitro* excitotoxic injury^36–39^.

This study corroborates our previous publication and establishes that the use of small molecule inhibitors is a robust approach for deriving neural crest cells from hESCs^20^. We observed a rapid downregulation of pluripotent genes (*NANOG* and *POU5F1*) and parallel induction of molecular markers (*NGFR*, *B3GAT1*, *POU4F1*, *TFAP2A, SNAI2*, *PRPH*, *NEFH*, *GFAP*, and *S100B*) in the NGFR^+^ population that was consistent with those of bona fide multipotent neural crest cells^40^. But despite the reproducibility of our experimental approach, it should be noted that one drawback of our methodology is the reliance on single marker cell sorting as previous studies have also noted^17,41,42^. We cannot therefore completely exclude the possibility that the isolated NGFR^+^ populations are composed of predominantly neural crest cells (cranial-, cardiac-, and trunk- subtypes) but also smaller populations of alternative dorsal neural tube derived cells. Eliminating this single marker enrichment step is therefore one of the critical obstacles that must be overcome if the routine preclinical use of these cells is to be realized^43^. This could be accomplished by transcriptome analyses of hESC-derived neural crest cell lines to identify novel signaling pathways that could subsequently be modulated during critical differentiation periods to promote the rapid and guided derivation of homogenous cell populations without the use of cell sorting^31^.

One important requirement for cell-based SCI therapy is that the transplanted cells must provide trophic support that fosters host tissue survival and regeneration^2,44^. Consistent with previous studies^45^ we observed that the differentiated cells expressed several trophic factors *in vitro* (*BDNF*, *NT-3*, *NGF*, *ANGPT1*, and *VEGFA*) with subsets of these transcripts being actively translated into proteins (BDNF, ANGPT1, and VEGFA) that were detectable in conditioned medium. Interestingly, negligible levels of NT-3 and NGF were once again observed that corroborated with our previous study^20^ and suggests that our differentiation approach yields a population of neural crest cells with a reproducible trophic factor profile. Moreover, these secreted trophic factors were biologically active and were able to induce robust neurite outgrowth of rat primary DRG neurons. Taken together, these results demonstrate that the neural crest cells generated in this study exhibited functional trophic factor profiles that are one of prerequisite traits for promoting neural regeneration and angiogenesis following SCI^46–48^.

The efficacy of the neural crest cells as preclinical therapy candidates was assessed in a rat cervical hemisection model of SCI. In contrast to contusion type of SCI with significant tissue necrosis and formation of cavities in the injury site, laceration cause by sharp bone fragments typically shows very limited cavitation^49^. In this study, the cavity was found in one animal with SCI alone and in one animal with cell transplantation. The observation that the S100B^+^ and NGFR^+^ transplanted cells were present within the injection sites and trauma zone 3 weeks after surgery is consistent with previous studies using xenogeneic stem cell transplantation with cyclosporine treatment^24,45^. Although cyclosporine has been found to be neuroprotective in an experimental traumatic brain and peripheral nerve injury models^25,50^, it seems to have differential effects upon transplanted stem cells^51^. Moreover, in compression SCI models the use of cyclosporine induces the upregulation of PDGF-A without any effect on other trophic factors^45^. Contrastingly, the neural crest cells derived in our study expressed elevated levels of *BDNF* in both ASCI and CSCI groups. Moreover, no teratoma formation was observed indicating the effective removal of pluripotent cells using our differentiation approach that concurs with our previous use of hESC-derived neural crest cells in a rat peripheral nerve injury model^20^.

The elevated *BDNF* levels observed in our study indicates the possibility that the regenerative effects of the transplanted cells in both ASCI and CSCI groups were mediated via neurotrophic support to different long spinal tracts. Numerous studies have demonstrated that BDNF has significant growth promoting and neuroprotective effects on bulbospinal tracts including 5-HT^+^ raphespinal axons and on sensory CGRP^+^ axons^46,52,53^. BDNF receptor TrkB is downregulated at the epicenter of SCI but remains largely unaffected in the segments rostral and caudal to the lesion site^54^. That these regions were utilized for cell transplantation in this study might account for the regenerative response observed in reply to the elevated levels of *BDNF* following transplantation. Although we did not find any signs of aberrant CGRP^+^ sprouting in the dorsal horn, the transplanted cells promoted ingrowth of 5-HT^+^ raphespinal axons deep into the trauma zone and induced their sprouting rostral to injury site. In addition, we found that the transplanted cells had no effects on the density of the reactive astrocytes in the scar but reduced their density both rostral and caudal to the trauma zone following CSCI which is in line with a recent report that human neural stem cells can attenuate reaction of astrocytes^55^. Although the mechanism is not completely understood, the authors demonstrate that the effect of stem cells on reactive astrocytes could be mediated via inhibition of NF-κB activity and associated immune response^55^. Unexpectedly, we did not find any effect of neural crest cells on host astrocytes following implantation into acutely injured spinal cord. This could possibly reflect the differences in tissue environment in the acutely and chronically injured spinal cord. Moreover, that the neural crest cells in the present study had no effects on neuroinflammation is in contrast to that recently observed for rat neural stem cells^56^.

Regaining upper limb function is of paramount importance to quadriplegic individuals following cervical SCI. Accordingly, axon regeneration for up to two spinal segments below the level of high C3–C4 cervical SCI would allow patients to regain control of both arm and shoulder muscles. To assess forelimb function, we used the vertical cylinder test that is known to be sensitive to the assessment of chronic limb use asymmetries^34^. It has been shown that this test is not influenced by repeated analysis and does not depend on aversive motivation^34^. Several reports suggest that injury to the lateral funiculus of the cervical spinal cord results in severe limb use asymmetries during rearing and landing without significant recovery in CSCI^34,57,58^. In contrast to single food pellet reaching test, which is often used to study fine motor control following cervical SCI, the vertical cylinder test does not require animal training before the injury. It has been also found that the outcome of the reaching test can be significantly affected by the differences in the rate of learning a motor task and different compensatory reaching strategies used by experimental animals with SCI^59^. We observed that neural crest cell transplantation progressively improved forelimb use for landing in both ASCI and CSCI groups. It is unlikely however that our observed improvement of locomotor function was due to axon regeneration across the injury site. Transplantation-induced sprouting of 5-HT^+^ raphespinal axons rostral to the injury site could indicate some form of neuronal relay formation between descending motor pathways in the lateral funiculus and propriospinal relay neurons^2,9^. Such a circuit could therefore transmit the signal to the neurons caudal to the injury site. It has been shown that human glial restricted progenitors could be very efficient in promoting axon regeneration of reticulospinal and raphespinal axons^60^ and the recovery mechanism of novel relay formation with propriospinal neurons has been reported previously for the corticospinal and reticulospinal tracts after incomplete cervical SCI^9,61,62,63^. Similar mechanisms could therefore account for the partial recovery of forelimb motor function observed in our study.

## Supplemental Material

Supplemental Material, sj-pdf-1-cll-10.1177_0963689720988245 - Human Embryonic Stem Cell–derived Neural Crest Cells Promote Sprouting and Motor Recovery Following Spinal Cord Injury in Adult Rats Click here for additional data file. Supplemental Material, sj-pdf-1-cll-10.1177_0963689720988245 for Human Embryonic Stem Cell–derived Neural Crest Cells Promote Sprouting and Motor Recovery Following Spinal Cord Injury in Adult Rats by Iwan Jones, Liudmila N. Novikova, Mikael Wiberg, Leif Carlsson and Lev N. Novikov in Cell Transplantation

## References

[1] Dvorak MF Noonan VK Fallah N Fisher CG Rivers CS Ahn H Tsai EC Linassi AG Christie SD Attabib N Hurlbert RJ, et al. Minimizing errors in acute traumatic spinal cord injury trials by acknowledging the heterogeneity of spinal cord anatomy and injury severity: an observational Canadian cohort analysis. J Neurotrauma. 2014;31(18):1540–1547. 24811484 10.1089/neu.2013.3278 PMC4161054

[2] Assinck P Duncan GJ Hilton BJ Plemel JR Tetzlaff W Cell transplantation therapy for spinal cord injury. Nat Neurosci. 2017;20(5):637–647. 28440805 10.1038/nn.4541

[3] Singh A Tetreault L Kalsi-Ryan S Nouri A Fehlings MG Global prevalence and incidence of traumatic spinal cord injury. Clin Epidemiol. 2014;6:309–331. 25278785 10.2147/CLEP.S68889 PMC4179833

[4] Houle JD Tessler A Repair of chronic spinal cord injury. Exp Neurol. 2003;182(2):247–260. 12895437 10.1016/s0014-4886(03)00029-3

[5] Tran AP Warren PM Silver J The biology of regeneration failure and success after spinal cord injury. Physiol Rev. 2018;98(2):881–917. 29513146 10.1152/physrev.00017.2017 PMC5966716

[6] Siddiqui AM Khazaei M Fehlings MG Translating mechanisms of neuroprotection, regeneration, and repair to treatment of spinal cord injury. Prog Brain Res. 2015;218:15–54. 25890131 10.1016/bs.pbr.2014.12.007

[7] Goritz C Dias DO Tomilin N Barbacid M Shupliakov O Frisen J A pericyte origin of spinal cord scar tissue. Science. 2011;333(6039):238–242. 21737741 10.1126/science.1203165

[8] Brennan FH Popovich PG Emerging targets for reprograming the immune response to promote repair and recovery of function after spinal cord injury. Curr Opin Neurol. 2018;31(3):334–344. 29465433 10.1097/WCO.0000000000000550

[9] Courtine G Sofroniew MV Spinal cord repair: advances in biology and technology. Nat Med. 2019;25(6):898–908. 31160817 10.1038/s41591-019-0475-6

[10] Pereira IM Marote A Salgado AJ Silva NA Filling the gap: neural stem cells as a promising therapy for spinal cord injury. Pharmaceuticals (Basel). 2019;12(2):65. 10.3390/ph12020065 PMC6631328 31035689

[11] Tetzlaff W Okon EB Karimi-Abdolrezaee S Hill CE Sparling JS Plemel JR Plunet WT Tsai EC Baptiste D Smithson LJ Kawaja MD, et al. A systematic review of cellular transplantation therapies for spinal cord injury. J. Neurotrauma. 2011;28(8):1611–1682. 20146557 10.1089/neu.2009.1177 PMC3143488

[12] Bunge MB Efficacy of Schwann cell transplantation for spinal cord repair is improved with combinatorial strategies. J Physiol. 2016;594(13):3533–3538. 26876753 10.1113/JP271531 PMC4929312

[13] Tsuji O Sugai K Yamaguchi R Tashiro S Nagoshi N Kohyama J Iida T Ohkubo T Itakura G Isoda M Shinozaki M, et al. Concise review: laying the groundwork for a first-in-human study of an induced pluripotent stem cell-based intervention for spinal cord injury. Stem Cells. 2019;37(1):6–13. 30371964 10.1002/stem.2926 PMC7379555

[14] Fairbairn NG Meppelink AM Ng-Glazier J Randolph MA Winograd JM Augmenting peripheral nerve regeneration using stem cells: a review of current opinion. World J Stem Cells. 2015;7(1):11–26. 25621102 10.4252/wjsc.v7.i1.11 PMC4300921

[15] Hofstetter CP Holmstrom NA Lilja JA Schweinhardt P Hao J Spenger C Wiesenfeld-Hallin Z Kurpad SN Frisen J Olson L Allodynia limits the usefulness of intraspinal neural stem cell grafts; directed differentiation improves outcome. Nat Neurosci. 2005;8(3):346–353. 15711542 10.1038/nn1405

[16] Steward O Sharp KG Yee KM Hatch MN Bonner JF Characterization of ectopic colonies that form in widespread areas of the nervous system with neural stem cell transplants into the site of a severe spinal cord injury. J Neurosci. 2014;34(42):14013–14021. 25319698 10.1523/JNEUROSCI.3066-14.2014 PMC4198542

[17] Lee G Kim H Elkabetz Y Al Shamy G Panagiotakos G Barberi T Tabar V Studer L Isolation and directed differentiation of neural crest stem cells derived from human embryonic stem cells. Nat Biotechnol. 2007;25(12):1468–1475. 18037878 10.1038/nbt1365

[18] Mujtaba T Mayer-Proschel M Rao MS A common neural progenitor for the CNS and PNS. Dev Biol. 1998;200(1):1–15. 9698451 10.1006/dbio.1998.8913

[19] Neirinckx V Cantinieaux D Coste C Rogister B Franzen R Wislet-Gendebien S Concise review: spinal cord injuries: how could adult mesenchymal and neural crest stem cells take up the challenge? Stem Cells. 2014;32(4):829–843. 24155224 10.1002/stem.1579

[20] Jones I Novikova LN Novikov LN Renardy M Ullrich A Wiberg M Carlsson L Kingham PJ Regenerative effects of human embryonic stem cell-derived neural crest cells for treatment of peripheral nerve injury. J Tissue Eng Regen Med. 2018;12(4):e2099–e2109. 29327452 10.1002/term.2642 PMC5947619

[21] Jozefczuk J Drews K Adjaye J Preparation of mouse embryonic fibroblast cells suitable for culturing human embryonic and induced pluripotent stem cells. J Vis Exp. 2012;21(64):3854. 10.3791/3854 PMC3471299 22760161

[22] Oh SK Kim HS Park YB Seol HW Kim YY Cho MS Ku SY Choi YM Kim DW Moon SY Methods for expansion of human embryonic stem. Stem Cells. 2005;23(5):605–609. 15849167 10.1634/stemcells.2004-0297

[23] Chambers SM Mica Y Lee G Studer L Tomishima MJ Dual-smad inhibition/wnt activation-based methods to induce neural crest and derivatives from human pluripotent stem cells. Methods Mol Biol. 2016;1307:329–343. 24301074 10.1007/7651_2013_59

[24] Kolar MK Kingham PJ Novikova LN Wiberg M Novikov LN The therapeutic effects of human adipose-derived stem cells in a rat cervical spinal cord injury model. Stem Cells Dev. 2014;23(14):1659–1674. 24803143 10.1089/scd.2013.0416

[25] McGrath A Brohlin M Wiberg R Kingham P Novikov LN Wiberg M Novikova LN Long-term effects of fibrin conduit with human mesenchymal stem cells and immunosuppression after peripheral nerve repair in a xenogenic model. Cell Medicine. 2018;10:1–13. 10.1177/2155179018760327 PMC6172997 32634185

[26] Jones I Yelhekar TD Wiberg R Kingham PJ Johansson S Wiberg M Carlsson L Development and validation of an *in vitro* model system to study peripheral sensory neuron development and injury. Sci Rep. 2018;8(1):15961. 30374154 10.1038/s41598-018-34280-3 PMC6206093

[27] Schindelin J Arganda-Carreras I Frise E Kaynig V Longair M Pietzsch T Preibisch S Rueden C Saalfeld S Schmid B Tinevez JY, et al. Fiji: an open-source platform for biological-image analysis. Nat Methods. 2012;9(7):676–682. 22743772 10.1038/nmeth.2019 PMC3855844

[28] Schaar KL Brenneman MM Savitz SI Functional assessments in the rodent stroke model. Exp Transl Stroke Med. 2010;2(1):13. 20642841 10.1186/2040-7378-2-13 PMC2915950

[29] Xu RH Sampsell-Barron TL Gu F Root S Peck RM Pan G Yu J Antosiewicz-Bourget J Tian S Stewart R Thomson JA NANOG is a direct target of TGFbeta/activin-mediated SMAD signaling in human ESCs. Cell Stem Cell. 2008;3(2):196–206. 18682241 10.1016/j.stem.2008.07.001 PMC2758041

[30] Noisa P Lund C Kanduri K Lund R Lahdesmaki H Lahesmaa R Lundin K Chokechuwattanalert H Otonkoski T Tuuri T Raivio T Notch signaling regulates the differentiation of neural crest from human pluripotent stem cells. J Cell Sci. 2014;127(Pt 9):2083–2094. 24569875 10.1242/jcs.145755

[31] Kreitzer FR Salomonis N Sheehan A Huang M Park JS Spindler MJ Lizarraga P Weiss WA So PL Conklin BR A robust method to derive functional neural crest cells from human pluripotent stem cells. Am J Stem Cells. 2013;2(2):119–131. 23862100 PMC3708511

[32] Usoskin D Furlan A Islam S Abdo H Lonnerberg P Lou D Hjerling-Leffler J Haeggstrom J Kharchenko O Kharchenko PV Linnarsson S, et al. Unbiased classification of sensory neuron types by large-scale single-cell RNA sequencing. Nat Neurosci. 2015;18(1):145–153. 25420068 10.1038/nn.3881

[33] Ramon-Cueto A Cordero MI Santos-Benito FF Avila J Functional recovery of paraplegic rats and motor axon regeneration in their spinal cords by olfactory ensheathing glia. Neuron. 2000;25(2):425–435. 10719896 10.1016/s0896-6273(00)80905-8

[34] Schallert T Fleming SM Leasure JL Tillerson JL Bland ST CNS plasticity and assessment of forelimb sensorimotor outcome in unilateral rat models of stroke, cortical ablation, Parkinsonism and spinal cord injury. Neuropharmacology. 2000;39(5):777–787. 10699444 10.1016/s0028-3908(00)00005-8

[35] Dupin E Sommer L Neural crest progenitors and stem cells: from early development to adulthood. Dev Biol. 2012;366(1):83–95. 22425619 10.1016/j.ydbio.2012.02.035

[36] Schizas N Konig N Andersson B Vasylovska S Hoeber J Kozlova EN Hailer NP Neural crest stem cells protect spinal cord neurons from excitotoxic damage and inhibit glial activation by secretion of brain-derived neurotrophic factor. Cell Tissue Res. 2018;372(3):493–505. 29516218 10.1007/s00441-018-2808-z PMC5949140

[37] Sieber-Blum M Schnell L Grim M Hu YF Schneider R Schwab ME Characterization of epidermal neural crest stem cell (EPI-NCSC) grafts in the lesioned spinal cord. Mol Cell Neurosci. 2006;32(1-2):67–81. 16626970 10.1016/j.mcn.2006.02.003

[38] Hoeber J Trolle C Konig N Du Z Gallo A Hermans E Aldskogius H Shortland P Zhang SC Deumens R Kozlova EN Human embryonic stem cell-derived progenitors assist functional sensory axon regeneration after dorsal root avulsion injury. Sci Rep. 2015;5:10666. 26053681 10.1038/srep10666 PMC4459081

[39] Neirinckx V Agirman G Coste C Marquet A Dion V Rogister B Franzen R Wislet S Adult bone marrow mesenchymal and neural crest stem cells are chemoattractive and accelerate motor recovery in a mouse model of spinal cord injury. Stem Cell Res Ther. 2015;6:211. 26530515 10.1186/s13287-015-0202-2 PMC4632651

[40] Bronner-Fraser M Neural crest cell formation and migration in the developing embryo. FASEB J. 1994;8(10):699–706. 8050668 10.1096/fasebj.8.10.8050668

[41] Jiang X Gwye Y McKeown SJ Bronner-Fraser M Lutzko C Lawlor ER Isolation and characterization of neural crest stem cells derived from *in vitro*-differentiated human embryonic stem cells. Stem Cells Dev. 2009;18(7):1059–1070. 19099373 10.1089/scd.2008.0362 PMC4606969

[42] Zhou Y Snead ML Derivation of cranial neural crest-like cells from human embryonic stem cells. Biochem Biophys Res Commun. 2008;376(3):542–547. 18804450 10.1016/j.bbrc.2008.09.032 PMC2574922

[43] Menendez L Yatskievych TA Antin PB Dalton S Wnt signaling and a Smad pathway blockade direct the differentiation of human pluripotent stem cells to multipotent neural crest cells. Proc Natl Acad Sci U S A. 2011;108(48):19240–19245. 22084120 10.1073/pnas.1113746108 PMC3228464

[44] Shroff G Gupta R Human embryonic stem cells in the treatment of patients with spinal cord injury. Ann Neurosci. 2015;22(4):208–216. 26526627 10.5214/ans.0972.7531.220404 PMC4627203

[45] Hawryluk GW Mothe A Wang J Wang S Tator C Fehlings MG An *in vivo* characterization of trophic factor production following neural precursor cell or bone marrow stromal cell transplantation for spinal cord injury. Stem Cells Dev. 2012;21(12):2222–2238. 22085254 10.1089/scd.2011.0596 PMC3411361

[46] Hollis ER Tuszynski MH Neurotrophins: potential therapeutic tools for the treatment of spinal cord injury. Neurotherapeutics. 2011;8(4):694–703. 21904786 10.1007/s13311-011-0074-9 PMC3250295

[47] Widenfalk J Lipson A Jubran M Hofstetter C Ebendal T Cao Y Olson L Vascular endothelial growth factor improves functional outcome and decreases secondary degeneration in experimental spinal cord contusion injury. Neuroscience. 2003;120(4):951–960. 12927201 10.1016/s0306-4522(03)00399-3

[48] Yu S Yao S Wen Y Wang Y Wang H Xu Q Angiogenic microspheres promote neural regeneration and motor function recovery after spinal cord injury in rats. Sci Rep. 2016;6:33428. 27641997 10.1038/srep33428 PMC5027575

[49] Harper GP Banyard PJ Sharpe PC The International Spinal Research Trust’s strategic approach to the development of treatments for the repair of spinal cord injury. Spinal Cord. 1996;34(8):449–459. 8856851 10.1038/sc.1996.78

[50] Sullivan PG Sebastian AH Hall ED Therapeutic window analysis of the neuroprotective effects of cyclosporine a after traumatic brain injury. J Neurotrauma. 2011;28(2):311–318. 21142667 10.1089/neu.2010.1646 PMC3037811

[51] Antonios JP Farah GJ Cleary DR Martin JR Ciacci JD Pham MH Immunosuppressive mechanisms for stem cell transplant survival in spinal cord injury. Neurosurg Focus. 2019;46(3):E9. 10.3171/2018.12.FOCUS18589 30835678

[52] Lu P Tuszynski MH Growth factors and combinatorial therapies for CNS regeneration. Exp Neurol. 2008;209(2):313–320. 17927983 10.1016/j.expneurol.2007.08.004 PMC2408882

[53] Weishaupt N Blesch A Fouad K BDNF: the career of a multifaceted neurotrophin in spinal cord injury. Exp Neurol. 2012;238(2):254–264. 22982152 10.1016/j.expneurol.2012.09.001

[54] Gerin CG Madueke IC Perkins T Hill S Smith K Haley B Allen SA Garcia RP Paunesku T Woloschak G Combination strategies for repair, plasticity, and regeneration using regulation of gene expression during the chronic phase after spinal cord injury. Synapse. 2011;65(12):1255–1281. 21308793 10.1002/syn.20903

[55] Karova K Wainwright JV Machova-Urdzikova L Pisal RV Schmidt M Jendelova P Jhanwar-Uniyal M Transplantation of neural precursors generated from spinal progenitor cells reduces inflammation in spinal cord injury via NF-kappaB pathway inhibition. J Neuroinflammation. 2019;16(1):12. 30654804 10.1186/s12974-019-1394-7 PMC6335809

[56] Sankavaram SR Hakim R Covacu R Frostell A Neumann S Svensson M Brundin L Adult neural progenitor cells transplanted into spinal cord injury differentiate into oligodendrocytes, enhance myelination, and contribute to recovery. Stem Cell Reports. 2019;12(5):950–966. 31031190 10.1016/j.stemcr.2019.03.013 PMC6524946

[57] Webb AA Muir GD Unilateral dorsal column and rubrospinal tract injuries affect overground locomotion in the unrestrained rat. Eur J Neurosci. 2003;18(2):412–422. 12887423 10.1046/j.1460-9568.2003.02768.x

[58] Liu Y Kim DH Himes BT Chow SY Schallert T Murray M Tessler A Fischer I Transplants of fibroblasts genetically modified to express BDNF promote regeneration of adult rat rubrospinal axons and recovery of forelimb function. J Neurosci. 1999;19(11):4370–4387. 10341240 10.1523/JNEUROSCI.19-11-04370.1999 PMC6782629

[59] Fouad K Hurd C Magnuson DS Functional testing in animal models of spinal cord injury: not as straight forward as one would think. Front Integr Neurosci. 2013;7:85. 24324414 10.3389/fnint.2013.00085 PMC3840303

[60] Jin Y Shumsky JS Fischer I Axonal regeneration of different tracts following transplants of human glial restricted progenitors into the injured spinal cord in rats. Brain Res. 2018;1686:101–112. 29408659 10.1016/j.brainres.2018.01.030 PMC5862559

[61] Weishaupt N Hurd C Wei DZ Fouad K Reticulospinal plasticity after cervical spinal cord injury in the rat involves withdrawal of projections below the injury. Exp Neurol. 2013;247C:241–249. 10.1016/j.expneurol.2013.05.003 23684634

[62] Filli L Engmann AK Zorner B Weinmann O Moraitis T Gullo M Kasper H Schneider R Schwab ME Bridging the gap: a reticulo-propriospinal detour bypassing an incomplete spinal cord injury. J Neurosci. 2014;34(40):13399–13410. 25274818 10.1523/JNEUROSCI.0701-14.2014 PMC6608315

[63] Bareyre FM Kerschensteiner M Raineteau O Mettenleiter TC Weinmann O Schwab ME The injured spinal cord spontaneously forms a new intraspinal circuit in adult rats. Nat Neurosci. 2004;7(3):269–277. 14966523 10.1038/nn1195

