# Supplementary material for: Human Embryonic Stem Cell–derived Neural Crest Cells Promote Sprouting and Motor Recovery Following Spinal Cord Injury in Adult Rats
Source: Cell Transplant. 2021 Jan 31;30:0963689720988245. doi: 10.1177/0963689720988245 (PMC7863557; doi:10.1177/0963689720988245)
Supplement: Supplemental Material, sj-pdf-1-cll-10.1177_0963689720988245 - Human Embryonic Stem Cell–derived Neural Crest Cells Promote Sprouting and Motor Recovery Following Spinal Cord Injury in Adult Rats [file sj-pdf-1-cll-10.1177_0963689720988245.pdf]

SUPPLEMENTARY INFORMATION

Human embryonic stem cell-derived neural crest cells promote sprouting and motor recovery following spinal cord injury in adult rats.

Iwan Jones[1,2], Liudmila N. Novikova[2], Mikael Wiberg[2,3], Leif Carlsson[1] & Lev N. Novikov[2*].

[1]Umeå Center for Molecular Medicine, Umeå University, SE-901 87 Umeå, Sweden; [2]Department of Integrative Medical Biology, Umeå University, SE-901 87 Umeå, Sweden; [3]Department of Surgical and Perioperative Science, Section of Hand and Plastic Surgery, Umeå University, SE-901 87 Umeå, Sweden.

[*]**Corresponding Author:** Lev N. Novikov, Department of Integrative Medical Biology, Umeå University, 901 87 Umeå, Sweden. Tel: +46 90 7869231; email: lev.novikov@umu.se

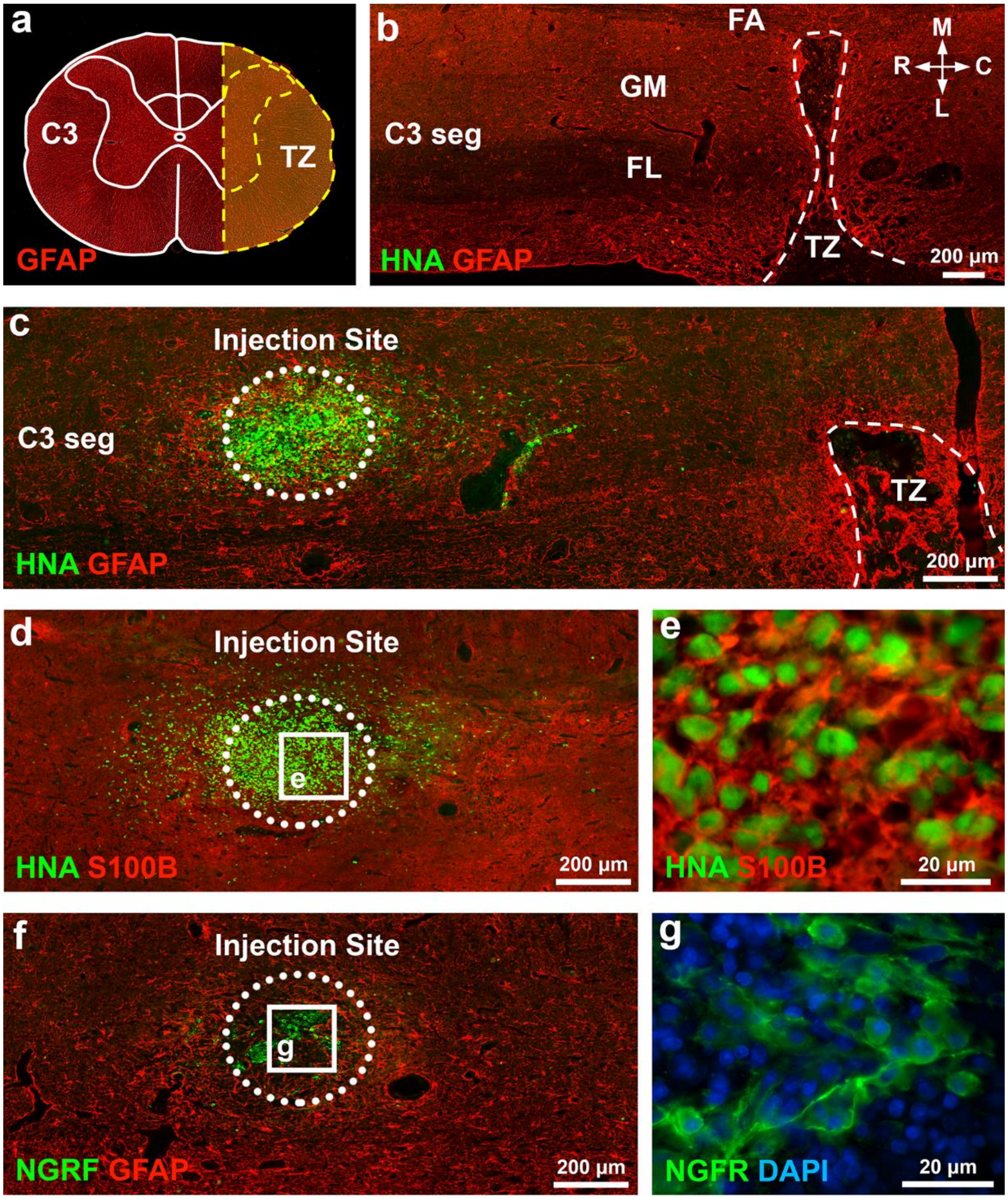

Supplementary Figure S1. Transplantation of differentiated neural crest cells into a rat chronic cervical spinal cord injury model. (a) Schematic diagram showing cervical spinal cord injury (SCI) model. (b) Representative horizontal C3 – C4 spinal cord section demonstrating control SCI at 3 weeks following vehicle injection. (c – g) Representative serial horizontal C3 – C4 CSCI sections demonstrating (c) HNA$^+$ neural crest cells, (d – e)

HNA^+ neural crest cells labelled for $S100B^+$ and (f – g) $NGFR^+$ cells. The image in (g) is counterstained with DAPI. Dotted circles indicate the injection sites and the dashed line indicates the trauma zone border. Note that in CSCI, neural crest cell transplantation did not result in a significant spread of injected cells along the rostro-caudal axis of the spinal cord. In contrast, the pressure of neural crest cell injection in ASCI displaced host astrocytes and the transplanted cells formed narrow tracts connected to the injury site (Figure 2). Scale bar: (b – d, f) 200 µm; (e, g) 20 µm. Abbreviations: ASCI, acute spinal cord injury; C, caudal; C3 seg, cervical spinal cord segment 3; CSCI, cervical spinal cord injury; DAPI, 4′,6-diamidino-2-phenylindole; FA, anterior funiculus, FL, lateral funiculus, GM, grey matter; L, lateral; M, medial; R, rostral; TZ, trauma zone.

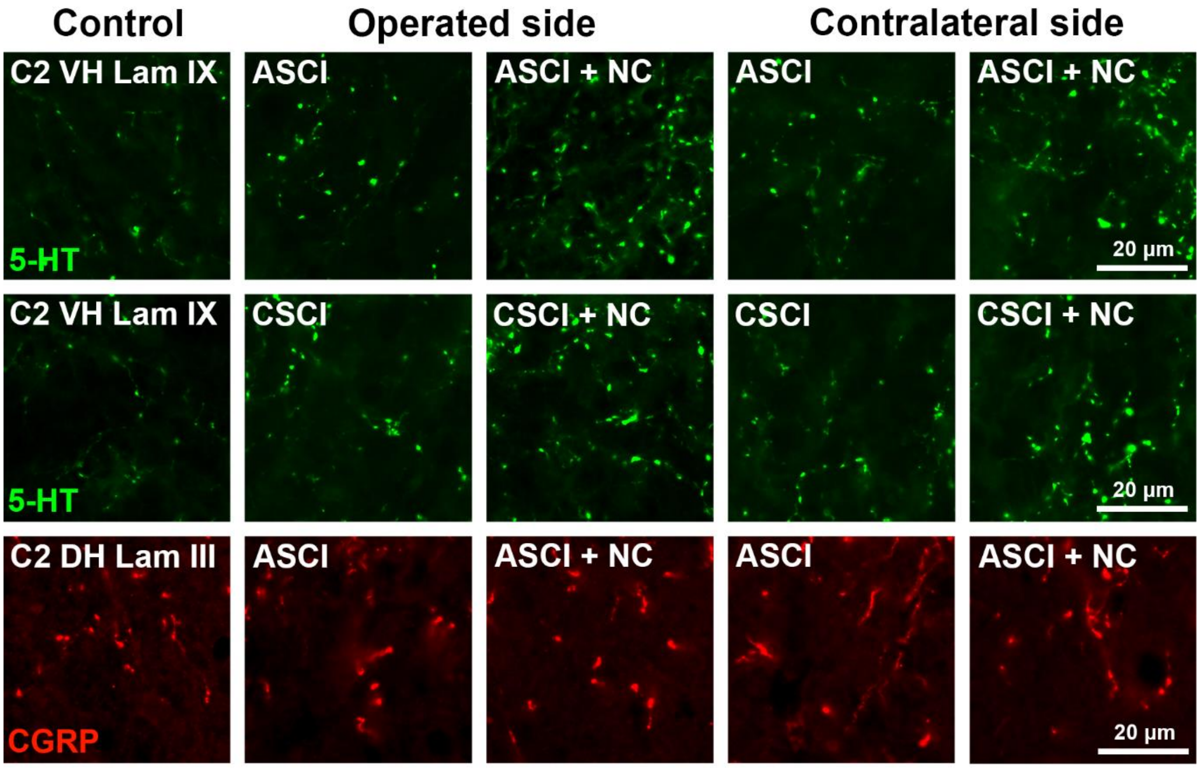

Supplementary Figure S2. The effects of neural crest cell transplantation on axonal sprouting. Representative transverse C2 spinal cord sections taken from control animals, animals at 16 – 23 weeks after spinal cord injury (ASCI, CSCI) and at 16 weeks following neural crest cell transplantation (ASCI +NC, CSCI +NC) demonstrating descending 5-HT$^+$ raphaespinal sprouting in Rexed lamina IX of the ventral horn and aberrant CGRP$^+$ sensory terminals in Rexed lamina III of the dorsal horn. Scale bar: 20 µm. Abbreviations: ASCI, acute spinal cord injury; C2, cervical spinal segment 2; CSCI, chronic spinal cord injury; DH, dorsal horn; Lam, Rexed lamina in the gravy matter of spinal cord; ASCI/CSCI +NC, spinal cord injury plus neural crest cell transplantation; VH, ventral horn.

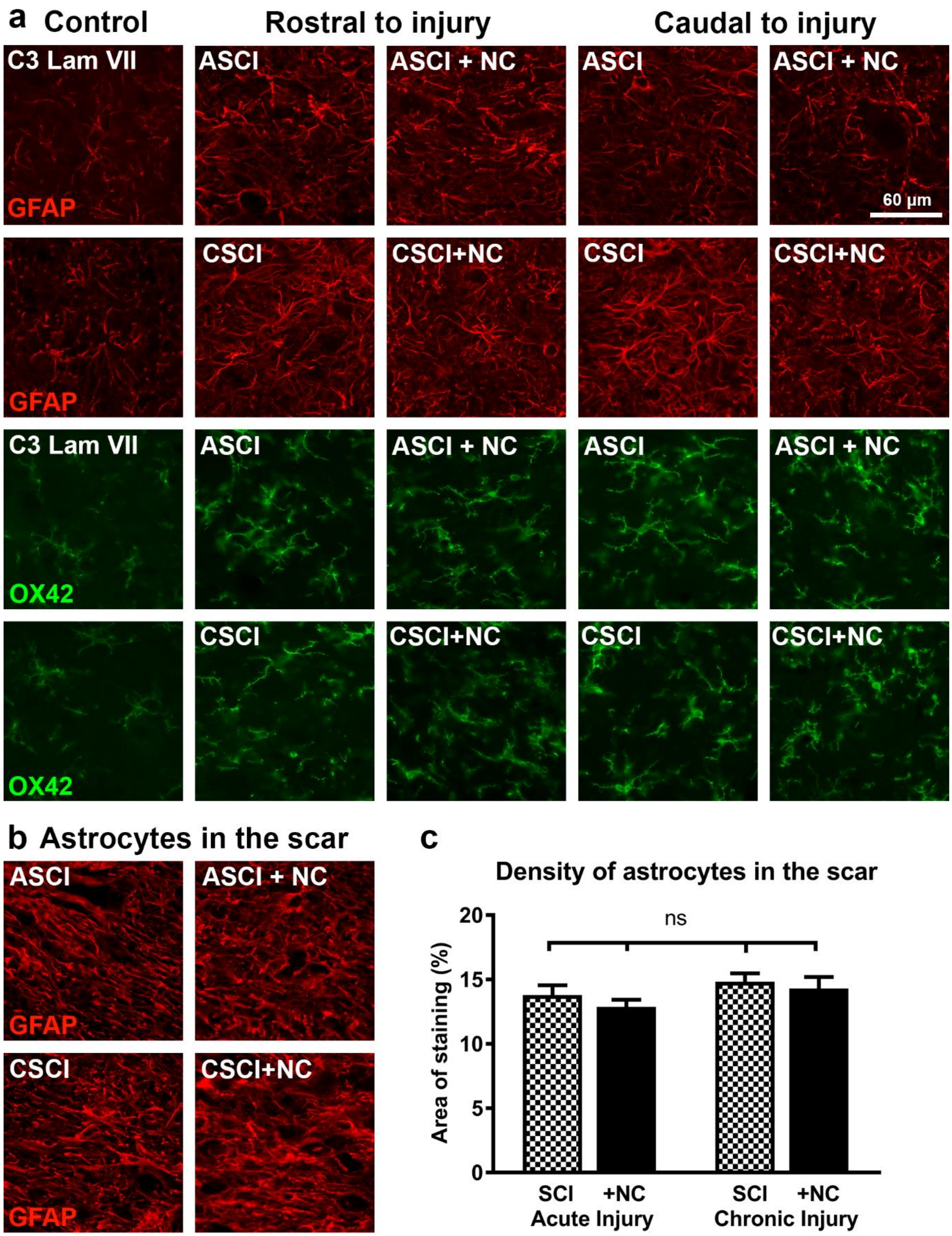

a Control
Rostral to injury
Caudal to injury
C3 Lam VII
ASCI
ASCI + NC
ASCI
ASCI + NC
GFAP
60 µm
CSCI
CSCI+NC
CSCI
CSCI+NC
GFAP
C3 Lam VII
ASCI
ASCI + NC
ASCI
ASCI + NC
OX42
CSCI
CSCI+NC
CSCI
CSCI+NC
OX42
b Astrocytes in the scar
ASCI
ASCI + NC
GFAP
CSCI
CSCI+NC
GFAP
c
Density of astrocytes in the scar
ns
Area of staining (%)
0
5
10
15
20
SCI
+NC
Acute Injury
SCI
+NC
Chronic Injury

Supplementary Figure S3. The effects of neural crest cell transplantation on reactivity of astrocytes and microglial cells. (a) Representative horizontal spinal cord sections taken from control animals, animals at 16 – 23 weeks after spinal cord injury (ASCI, CSCI) and at 16 weeks following neural crest cell transplantation (ASCI +NC, CSCI +NC) both rostral and caudal to the injury site (2000 – 2250 μm) demonstrating reactive $GFAP^+$ astrocytes and $OX42^+$ microglial cells in lamina VII. Quantitative data is shown in Figure 6. (b) Representative horizontal spinal cord sections taken from control animals at 16 – 23 weeks after spinal cord injury (ASCI, CSCI) and at 16 weeks following neural crest cell transplantation (ASCI +NC, CSCI +NC) demonstrating reactive $GFAP^+$ astrocytes in the scar tissue surrounding the injury site. (c) Histogram showing the density of $GFAP^+$ astrocytes in the scar. All data represents the mean ± SEM. Statistical significances were calculated using a one-way analysis of variance (ANOVA) followed by Tukey's multiple comparisons test using data derived from ASCI (n = 5), ASCI +NC (n = 5), CSCI (n = 5) and CSCI +NC (n = 5) groups. Scale bar: 60 μm. Abbreviations: ASCI, acute spinal cord injury; C3, cervical spinal segment 3; CSCI, chronic spinal cord injury; Lam, Rexed lamina in the gravy matter of spinal cord; ASCI/CSCI +NC, spinal cord injury plus neural crest cell transplantation.

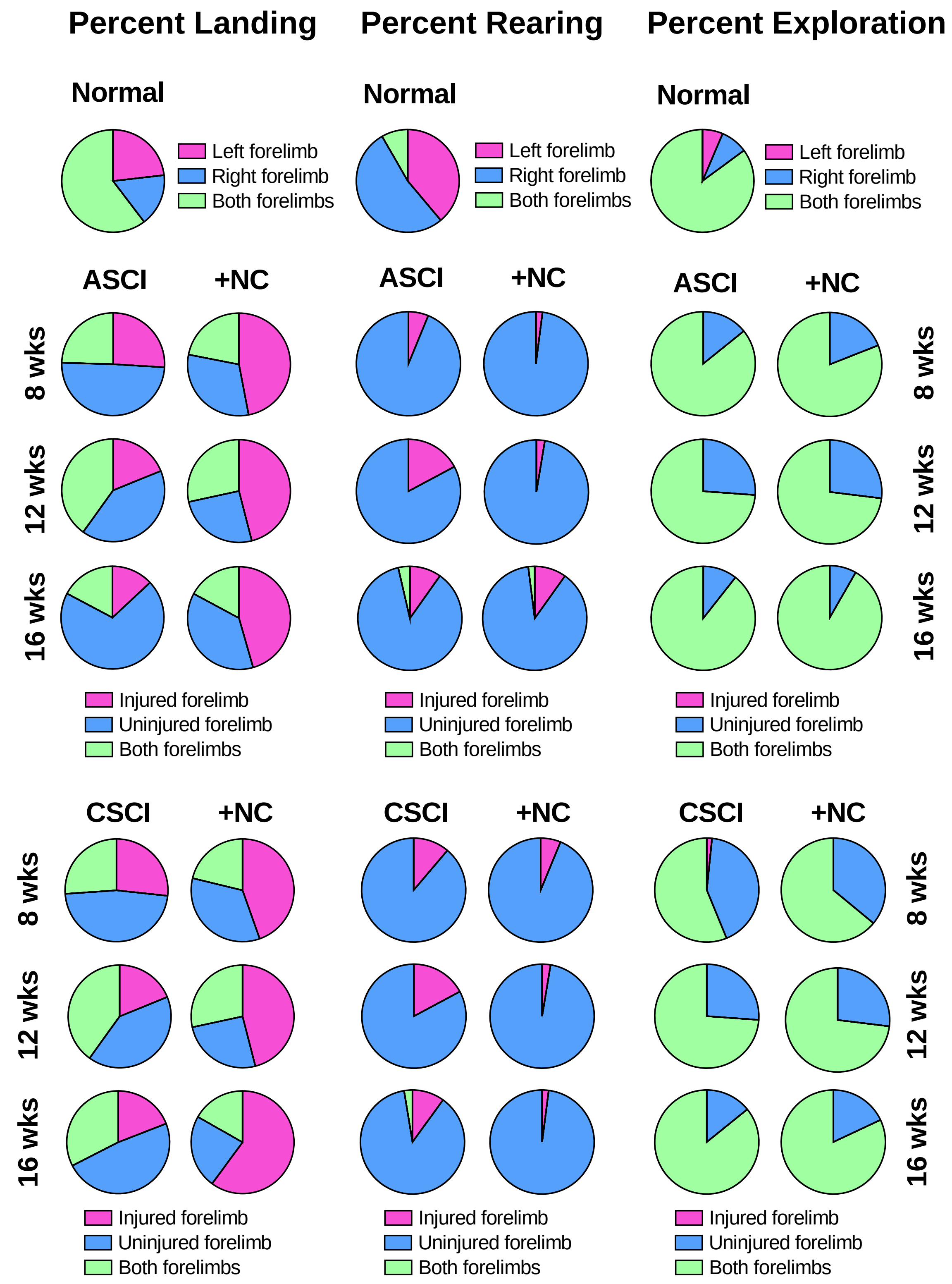
Percent Landing
Percent Rearing
Percent Exploration
Normal
Normal
Normal
Left forelimb
Right forelimb
Both forelimbs
Left forelimb
Right forelimb
Both forelimbs
Left forelimb
Right forelimb
Both forelimbs
ASCI
+NC
ASCI
+NC
ASCI
+NC
8 wks
12 wks
16 wks
8 wks
12 wks
16 wks
Injured forelimb
Uninjured forelimb
Both forelimbs
Injured forelimb
Uninjured forelimb
Both forelimbs
Injured forelimb
Uninjured forelimb
Both forelimbs
CSCI
+NC
CSCI
+NC
CSCI
+NC
8 wks
12 wks
16 wks
8 wks
12 wks
16 wks
Injured forelimb
Uninjured forelimb
Both forelimbs
Injured forelimb
Uninjured forelimb
Both forelimbs
Injured forelimb
Uninjured forelimb
Both forelimbs

Supplementary Figure S4. Neural crest cell transplantation improves landing and weight bearing activity following spinal cord injury. (a – b) Pie charts showing that neural crest cell transplantation improves the spontaneous use of the injured forelimb for landing and weight bearing in both ASCI and CSCI treatment groups compared to control SCI animals. Quantitative data for rearing and landing is shown in Figure 7. All data is derived from normal uninjured animals (*n* = 10), ASCI +NC (*n* = 7), ASCI (*n* = 5), CSCI +NC (*n* = 5) and CSCI (*n* = 5) groups. Abbreviations: ASCI acute spinal cord injury control, CSCI, chronic spinal cord injury control, +NC, treatment with neural crest cells; Normal, uninjured control;

Supplementary Table S1. Experimental groups and number of animals

Experimental group	Neurite outgrowth assay	qRT-PCR ELISA	IHH with qualitative analysis	Vertical cylinder test	IHH with quantitative analysis
Normal control	6			10	5
ASCI+NC, 3 weeks		5	5		
CSCI+NC, 3 weeks		4	3		
SCI control, 3 weeks		5	2		
ASCI+NC, 16 weeks				7*	7*
CSCI+NC, 16 weeks				5*	5*
SCI control, 16 weeks				5*	5*
CSCI alone, 23 weeks				5*	5*

* Experimental animals were first tested for recovery of forelimb motor function (8, 12 and 16 weeks after cell injection) and then sacrificed for quantitative analysis of neuronal and glial markers (16 weeks after cell injection).

Supplementary Table S2. **Species-specific oligonucleotide sequences used in this study.**

**Gene**	**Sense Primer (5′→3′)**	**Antisense Primer (5′→3′)**
hANGPT1	CTTGACCGTGAATCTGGAGC	AGCAAGACATAACAGGTGAG
hB3GAT1	CGGAAGCAGGTTTGGAGA	CGGAGACGCTCCGGACT
hBDNF	AGAGGCTTGACATCATTGGCTG	CAAAGGCACTTGACTACTGAGCATC
hNANOG	CTCAGCCTCCAGCAGATGC	TAGATTTCATTCTCTGGTTCTGG
hNFGR	CAGGCTTTGCAGCACTCAC	CTGCTGCTGTTGCTGCTTCT
hNGF	ATACAGGCGGAACCACACTCAG	GTCCACAGTAATGTTGCGGGTC
hNTF3	GGGAGATCAAAACGGGCAAC	ACAAGGCACACACACAGGAC
hPOU5F1	TTGGGCTCGAGAAGGATGTG	GTGAAGTGAGGGCTCCCATA
hSNAI2	GCAGTGAGGGCAAGAAAAAG	TCGGACCCACACATTACCTT
hTBP	CTTCCGCTGGCCCATAGTGA	CCAAGAAACAGTGATGCTGGGT
hVEGFA	TACCTCCACCATGCCAAGT	TGCATTCACATTTGTTGTGC
rBcan	CATCCAGAACCCACGAGAAG	GGCATAACAGTAGACATCGTAGAG
rNcan	CCAGAGATTCAGGCTTCGTAAA	CTGGTCCTCCACTCAAATACAG
rPtprz1	GAAGAGATTGGCTGGTCCTATAC	GGAGACTGCTTTGGGCTATT
rTBP	TCATGGTGCGTGACGATAAC	CTGGTCCATGACTCTCACTTTC
rVcan	ATCGTGTGGGCCATGATTAC	GTTGGGTCTCCAGTTCTCATATT

Supplementary Table S3. List of primary antibodies used in this study.

**Antibody**	**Dilution**	**Host Species**	**Manufacturer**	**Product Number**
5-HT	1:1000	Mouse	Dako	M0758
B3GAT1	1:100	Mouse	Sigma-Aldrich	C6680
αSMA	1:200	Mouse	Sigma-Aldrich	A5228
Thy-1/CD90	1:20	Mouse	Chemicon	CBL415
CGRP	1:1000	Rabbit	Chemicon	AB15360
Cytokeratin	1:200	Mouse	Novus	NBP2-29429
GFAP	1:1000	Rabbit	Dako	Z0334
Glut4	1:200	Rabbit	Novus	NBP1-49533
HNA	1:100	Mouse	Chemicon	MAB1281
MBP	1:500	Rabbit	Abcam	ab133620
NEFH	1:500	Mouse	Covance	SMI31R
NFGR	1:100	Mouse	Advanced Targeting Systems	ABN07
NeuN	1:200	Mouse	Chemicon	MAB377
OX42	1:250	Mouse	Serotec	MCA275G
POU4F1	1:500	Rabbit	Millipore	AB5945
PRPH	1:1000	Rabbit	Millipore	AB1530
S100B	1:2000	Rabbit	Dako	Z0311
TFAP2A	1:100	Mouse	DHSB	3B5
TUBB3	1:500	Rabbit	Abcam	ab18207
